# Bond market opening, monetary policy, and systemic financial risks – An empirical study based on the TVP-SV-VAR model

**DOI:** 10.1371/journal.pone.0335859

**Published:** 2025-11-03

**Authors:** Wei-Ying Ping, Yu-Wen Hu, Liang-Qing Luo

**Affiliations:** School of Statistics and Data Science, Jiangxi University of Finance and Economics, Nanchang, Jiangxi Province, China; Universiti Malaysia Sabah, MALAYSIA

## Abstract

While the opening of the bond market provides strong support for high-level financial opening, it also accelerates the accumulation of systemic financial risks, thereby affecting the high-quality development of China’s finance. Based on data from 2003 to 2024, this paper measures China’s bond market opening, monetary policy, and systemic financial risks, and employs a TVP-SV-VAR model to investigate the time-varying relationships among bond market opening, monetary policy, and systemic financial risks. The findings are as follows: (1) The impact of bond market opening on China’s systemic financial risks exhibits time-varying characteristics; (2) Contractionary monetary policy helps curb systemic financial risks, but this effect marginally diminishes when facing external structural shocks; (3) The improvement of interest rate transmission mechanisms and the transition toward price-based monetary policy can significantly enhance the sustainability of monetary policy’s regulatory role in systemic financial risks; (4) There exists a significant linkage effect between bond market opening and monetary policy, but this effect is subject to time-varying influences from the progress of domestic institutional reforms and cross-border capital anomalies.

## 1. Introduction

Against the backdrop of deepening global economic integration and cross-border financial market integration, high-level financial opening has become a core strategy for countries to enhance their international financial competitiveness, and it is also a key focus of academia and policymakers. The high-level meeting of the Chinese government in January 2024 clearly stated that institutional opening is the core path for high-level financial opening, providing top-level guidance for the opening of key financial markets such as bonds and stocks. Among these, the bond market — as the onshore financial market in China with the highest participation of international capital and the most mature marketization — its depth and breadth of opening directly affect the effectiveness of the financial opening strategy. It is also a key link connecting domestic and foreign capital markets and promoting the internationalization of the RMB. Statistics show that by the end of 2023, foreign investors held RMB 7.8 trillion of financial assets in China, with bond holdings exceeding RMB 3.2 trillion, accounting for over 40% of their RMB asset allocation [1]. This not only confirms the phased achievements of China’s bond market opening but also highlights its important value and strategic role in global capital allocation. Mechanically, bond market opening supports interest rate marketization reform and RMB internationalization by reducing cross-border financial transaction costs, optimizing bond risk pricing, and promoting market-oriented allocation of financial resources, making it a key driver for achieving high-quality financial development.

However, the fragility of emerging market financial systems exposes China’s bond market opening to the challenge of structural risks from cross-border capital flows. Frequent entry and exit of international short-term capital in the bond market and speculative activities may trigger violent fluctuations in cross-border capital, disrupting market liquidity and asset price stability. At the same time, cross-border capital flows in China currently exhibit new characteristics of expanded scale, complex structure, and normalized volatility. The existing regulatory framework has adaptability issues in risk identification, early warning, and disposal, leading to significant pressure on systemic financial risks prevention and control. Furthermore, in the process of bond market opening, monetary policy must balance the effectiveness of aggregate regulation and financial stability. Increased sensitivity of cross-border capital flows may reduce the transmission efficiency of monetary policy, and coordination frictions between macro-prudential policy and monetary policy may easily breach the risk threshold of the financial system and trigger systemic financial risks when risks from exchange rates, interest rates, and cross-border capital flows resonate—this requires focused attention from academia and policymakers.

A review of existing literature reveals that although scholars have explored bond market opening, monetary policy, and systemic financial risks, three gaps remain. First, most research frameworks are static: they either examine the impact of bond market opening on systemic financial risks in isolation or analyze the relationship between monetary policy and systemic financial risks independently, failing to integrate the three into a dynamic analytical framework. This makes it difficult to capture the time-varying characteristics of their relationships, limiting the applicability and timeliness of research conclusions. Second, the exploration of mechanisms lacks a time-varying perspective: existing studies do not systematically analyze the linkage effect between bond market opening and monetary policy, especially ignoring the combined effect of these two variables on systemic financial risks through channels such as cross-border capital flows, exchange rates, and interest rates, leading to an incomplete understanding of risk evolution paths. Third, the measurement of systemic financial risks often relies on non-time-varying weighting methods such as the entropy method and principal component analysis. Static weights cannot accurately reflect changes in the risk contribution of different sub-markets and risk factors in the financial system, failing to dynamically capture risk transmission paths and evolution trends, which undermines the accuracy and timeliness of measurement results.

Therefore, constructing a dynamic and unified analytical framework for bond market opening, monetary policy, and systemic financial risks is of urgent theoretical and practical significance. Theoretically, this framework can break the limitations of single-variable analysis, place the three variables in the same dynamic system, fully depict the linkage mechanism between bond market opening and monetary policy, and their transmission paths affecting systemic financial risks. This enables accurate capture of the time-varying characteristics of their relationships, making up for the deficiencies of existing studies in dynamics and systematisms and enriching the relevant theoretical system. Practically, it can provide a scientific analytical tool for systemic financial risks prevention, offer references for improving the macro-prudential management framework and promoting the orderly opening of the bond market, and help policymakers balance opening and risk prevention. This contributes to achieving high-quality financial development and safeguarding national financial security.

To address the above research background and gaps, the marginal contributions of this study are as follows: (1) Construct a scientific measurement system to accurately depict the dynamic levels of bond market opening, monetary policy intensity, and systemic financial risks through dynamic weighting methods; (2) Clarify the time-varying correlation mechanisms and transmission paths among bond market opening, monetary policy, and systemic financial risks, and reveal the evolutionary laws of their relationships in different market stages; (3) Analyze the specific channels through which the linkage effect of bond market opening and monetary policy affects systemic financial risks prevention, and identify risk amplification or mitigation mechanisms under different channels. Based on this, the research results can provide theoretical support for systemic financial risks prevention and practical references for improving the macro-prudential management framework and promoting the orderly opening of the bond market, which is of great significance for achieving high-quality financial development.

The remainder of this paper is structured as follows: Section 2 presents the literature review and theoretical analysis; Section 3 describes the model specification and data; Section 4 analyzes the empirical results; Section 5 discusses the relationship between governance quality and foreign direct investment through heterogeneity tests; and the paper concludes with Section 6.

## 2. Literature review and theoretical analysis

This section focuses on the relationships among bond market opening, monetary policy, and systemic financial risks, providing a systematic review and theoretical analysis of existing research results.

### 2.1 Research on the impact of bond market opening on systemic financial risks

Existing studies hold divergent views on this relationship. Some scholars argue that bond market opening exacerbates systemic financial risks: Zhao [2] points out that financial market opening exposes economies to international capital speculation and financial sanctions; Kim and Singal [3] focus on price risk, arguing that bond market opening amplifies exchange rate and interest rate volatility; Li and Li [4] and Zhao et al. [5] supplement this view by noting that foreign institutions may crowd out domestic entities and that regulatory coverage may be insufficient. Other scholars emphasize two-way effects: Wang [6] proposes that bond market opening forces enterprises to strengthen information disclosure robustness; Zhao et al. [7] note that bond market opening eases financing constraints; and Lu [8] argues that bond market opening restricts excessive corporate investment through external supervision.

The mechanism can be analyzed from three dimensions: cross-border capital flows, cross-border risk contagion, and market structure optimization. Under different mechanisms, the effects of exacerbating and mitigating systemic financial risks coexist, which are influenced by market systems and the maturity of supervision.

#### 2.1.1 Cross-border capital flow dimension.

Foreign capital can enhance bond market liquidity, improve yield curve pricing, and provide support for financial stability, but high volatility exacerbates financial fragility. Zhang and Xiao [9] pointed out that after the opening of emerging economies, foreign capital exhibits a “fast in and fast out” pattern. When global risk appetite declines or developed economies tighten monetary policy, concentrated capital outflows lead to bond price declines and yield jumps, increasing the pressure of asset depreciation on financial institutions and potentially triggering liquidity crises. Forbes and Warnock [10] verified that the degree of bond market opening is positively correlated with cross-border capital volatility, and the impact of abnormal volatility on emerging markets is stronger than that on mature markets.

#### 2.1.2 Cross-border risk contagion dimension.

The opening of the bond market strengthens the linkage between domestic and foreign finance, highlighting risk resonance and complex contagion paths. Cross-border asset allocation by foreign institutions transmits bond risks through the balance sheets of financial institutions, accelerating the spread of systemic financial risks. Li and Zhang [11] took the inclusion of China’s bond market in international indices as an example, pointing out that the risk spillover effect intensifies as the proportion of foreign holdings increases. During the 2008 financial crisis, fluctuations in U.S. Treasury bonds were transmitted to China through cross-border portfolios, causing abnormal short-term interest rate fluctuations in China. Fang [12] emphasized that under opening, cross-border risk contagion paths are more diversified, and the liquidation or rebalancing of positions by foreign institutions may amplify domestic volatility and exacerbate risk transmission.

#### 2.1.3 Market structure optimization dimension.

The opening of the bond market has a risk-mitigating effect, but it is constrained by defects in market systems. Positive aspects: The introduction of foreign investors improves the liquidity and pricing efficiency of the bond market, reduces bond issuance costs, and guides capital to concentrate on high-quality entities. It promotes the integration of China’s domestic bond market with international standards, and the “catfish effect” brought by foreign rating agencies forces the transformation of domestic credit rating practices. Li [13] indicates that the mitigation of inflated credit bond ratings in 2019 is associated with this effect. Additionally, the supervision mechanisms, default resolution systems, and risk hedging tools established during the opening process can consolidate the risk bottom line, buffer interest rate fluctuations, and inhibit the cross-market transmission of risks. Negative aspects: The immaturity of domestic bond market supervision—such as insufficient credibility of ratings, inefficient default resolution, and lack of derivatives—leads to liquidity stratification of low-rated bonds and increases market fragility. This is highly consistent with the conclusion proposed by Yang et al. [14] that “default resolution efficiency directly affects financial stability”.

### 2.2 Research on the impact of monetary policy on systemic financial risks

Academic circles generally agree that the relationship between the two is bidirectional and complex. In terms of tool effects: Guo et al. [15] find that quantitative tool shocks are more direct; He et al. [16] note that quantitative tools are effective in both short and long terms, while price-based tools are only effective in the short term. In terms of policy direction: Chen and Wu [17] confirm that tight policies increase capital costs; Ma [18] emphasizes that loose policies mitigate risk in the short term but accumulate risk in the long term.

The mechanism of monetary policy can be analyzed from two dimensions: interest rate and liquidity regulation, and financial institution behavior guidance. The regulatory effects and timeliness of different policy tools vary significantly, which are influenced by factors such as policy duration and economic environment.

#### 2.2.1 Interest rate and liquidity regulation dimension.

As the core channel, policies with different directions produce opposite effects. Easy monetary policy reduces the benchmark interest rate and increases liquidity in the short term, lowering corporate financing costs and mitigating risks, but it tends to push up leverage and fuel asset bubbles in the long term. Targeted easing may even exacerbate liquidity inequality, leading to capital diversion from the real economy to speculative activities. Bernanke [19] took the Federal Reserve’s practice from 2001 to 2004 as an example, pointing out that long-term low interest rates stimulated excessive leverage in the U.S. real estate sector, turning local risks into a global crisis.

#### 2.2.2 Financial institution behavior guidance dimension.

As an indirect channel, it functions by changing the operational decisions and risk preferences of financial institutions. In an easy monetary environment, long-term low interest rates compress interest margins. To maintain profits, financial institutions increase their risk appetite, expand holdings of high-risk assets, push up leverage ratios, and exacerbate risks. Adrian and Shin [20] confirmed that financial institutions engage in profit-seeking risk-taking under low interest rates, which increases the fragility of the financial system. In a tight monetary environment, higher interest rates increase operational costs, reduce arbitrage scale and leverage ratios, and force financial institutions to strengthen risk management and enhance risk resilience. Additionally, it guides them to return to prudent operations and redirect financial resources to the real economy, fundamentally reducing risk accumulation.

### 2.3 Research on the impact of monetary policy on bond market opening

Existing literature explores the impact of monetary policy on bond market opening from both domestic and international perspectives. Regarding the impact of domestic monetary policy on the bond market: Yao and Zheng [21] confirm that concentrated adjustments of monetary policy significantly exacerbate market liquidity volatility, especially emphasizing that price-based tools have a more direct and significant impact. Regarding the impact of international monetary policy on bond market opening: Chen and Liu [22] note that when domestic and foreign financial risks rise, the spillover effect and financial contagion of U.S. monetary policy on China’s bond market intensify, and the impact of tight policy shocks is more significant.

The mechanism of monetary policy on bond market opening mainly unfolds through two aspects: interest rate transmission effect and foreign exchange policy coordination.

#### 2.3.1 Interest rate transmission effect dimension.

Interest rate spreads drive cross-border capital flows: Taylor [23] proposed that monetary policy should adjust interest rates based on inflation and output gaps. As a core driver of cross-border capital flows, interest rate spreads directly affect foreign capital’s bond allocation decisions. For example, in 2020, the yield of Chinese Treasury bonds was 2.5 percentage points higher than that of U.S. Treasury bonds, driving the scale of foreign holdings of Chinese Treasury bonds to increase by 67% year-on-year. In 2022, the Federal Reserve’s interest rate hikes caused the China-U.S. interest rate spread to turn from positive to negative, leading foreign capital to reduce holdings of RMB-denominated bonds by nearly RMB 800 billion.

Policy interest rates guide market pricing: Ma [24] pointed out that interest rate marketization reform enhances foreign capital’s ability to manage expectations of long-term interest rates by improving the transmission efficiency and transparency of the yield curve. This attracts long-term capital inflows to obtain stable term spread returns, thereby increasing the scale of foreign capital’s allocation to Chinese Treasury bonds.

#### 2.3.2 Foreign exchange policy coordination dimension.

The central bank maintains the stability of the RMB exchange rate through foreign exchange intervention and expectation management, reducing foreign exchange risk for foreign capital and enhancing their confidence in allocating RMB-denominated bonds. In 2020, overseas institutions increased their holdings of RMB-denominated bonds by USD 59.6 billion, partly due to the expectation of RMB appreciation and the stable environment guided by exchange rate policies. This is consistent with the conclusion of Frankel and Wei [25] that “exchange rate stability promotes foreign capital inflows”.

### 2.4 Research on the impact of bond market opening on monetary policy

Existing literature explores the impact of bond market opening on monetary policy, with views focusing on different aspects. Liu and Su [26] found that bond market opening amplifies short-term shocks from foreign policies. Lü and Peng [27] proposed that bond market opening can buffer against exchange rate depreciation, but the effective play of this role requires the mitigation of depreciation expectations as a prerequisite. Wang et al. [28] pointed out that bond market opening will weaken the output effect of monetary policy.

The impact of bond market opening on monetary policy mainly operates through two dimensions: capital flow constraints and foreign exchange policy constraints.

#### 2.4.1 Capital flow constraint dimension.

The linkage between domestic and foreign interest rates and the interest rate parity mechanism cause foreign capital flows to significantly affect domestic interest rates. In 2022, the Federal Reserve’s aggressive interest rate hikes narrowed and inverted the China-U.S. Treasury bond spread, triggering large-scale selling of RMB-denominated bonds and increased holdings of U.S. Treasury bonds by foreign capital. This not only pushed up the yield of Chinese Treasury bonds (leading to asset depreciation) but also offset the effect of domestic interest rate cut policies during the same period through market interest rate transmission, weakening regulatory effects. The VAR model empirical study by Yu and Xiao [29] showed that the impact coefficient of foreign capital flows on China’s Treasury bond yields increases significantly with the improvement of bond market opening.

#### 2.4.2 Foreign exchange policy constraint dimension.

Bond market opening exacerbates the contradiction of the “Impossible Trinity”, restricting the autonomy and effectiveness of monetary policy. Under the Impossible Trinity, it is difficult to simultaneously achieve free capital flow, fixed exchange rate, and independent monetary policy. Bond market opening promotes cross-border capital flows, putting the central bank in a trade-off dilemma between “stabilizing the exchange rate” and “stabilizing interest rates”: large-scale inflows and outflows of foreign capital cause sharp exchange rate fluctuations. The central bank must either use foreign exchange reserves for intervention or suspend monetary policy adjustments to maintain interest rate spreads, ultimately weakening policy effectiveness. Additionally, Bruno and Shin [30] pointed out that foreign investors use derivatives to hedge exchange rate risks, and concentrated liquidation behavior during market volatility will exacerbate fluctuations, disrupt short-term policy interest rate transmission, and further weaken the effect of monetary policy.

## 3. Empirical model and data analysis

### 3.1. Empirical model

This study employs the time-varying parameter vector autoregressive model with stochastic volatility (TVP-SV-VAR) to analyze the time-varying relationship among the opening of the bond market, monetary policy and systemic financial risks. The TVP-SV-VAR model, proposed by Nakajima [31], gradually introduces time-varying parameters and stochastic volatility into the VAR framework, thus enabling dynamic analysis of time-varying characteristics and nonlinear relationships between variables. The mathematical expression of the standard VAR model is as follows:


$$y_{t}=A_{\text{1}}y_{t-\text{1}}+A_{\text{2}}y_{t-\text{2}}+\ldots +A_{p}y_{t-p}+\varepsilon_{t},\;{\;\varepsilon }_{t}\sim N(\text{0},\Sigma )\;$$ (1)

Where $y_{t}$ is n×1 dimensional variable vector,$A_{i}$ is the coefficient matrix, $\varepsilon_{t}$ is the error term, and the covariance matrix Σis constant.

The coefficient matrix and covariance matrix are extended to time-varying form:


$$y_{t}=A_{\text{1},t}y_{t-\text{1}}+A_{\text{2},t}y_{t-\text{2}}+\ldots +A_{p,t}y_{t-p}+\varepsilon_{t},\;\varepsilon_{t}\sim N(\text{0},\Sigma_{t})\;$$ (2)

Furthermore, the coefficient matrix is stacked as$\beta_{t}=vec\left({\left[A_{\text{1},t},\ldots ,A_{p,t}\right]}^{\prime }\right)$, and the model can be rewritten as a state space form:


$$y_{t}=X_{t}\beta_{t}+\varepsilon_{t}$$ (3)

Where $X_{t}=I_{k}⊗{\left[{y^{\prime }}_{t-\text{1}},\ldots ,{y^{\prime }}_{t-p}\right]}^{\prime }$, ⊗ refers to Kronecker.


$$\beta_{t}=\beta_{t-\text{1}}+\mu_{t},\;\mu_{t}\sim N(\text{0},Q)$$ (4)

Where $\mu_{t}$ is the parameter perturbation term and Q is the covariance matrix. Further, the covariance matrix $\Sigma_{t}$ is decomposed into time-varying volatility and correlation coefficient matrix by Cholesky decomposition method,


$$\Sigma_{t}=L_{t}DL_{t}^{\prime }\;$$ (5)

where $\;L_{t}=diag(\sigma_{\text{1},t},\ldots ,\sigma_{p,t})$, $\sigma_{i,t}$ represents the time-varying standard deviation of the ith variable; Dis the constant correlation coefficient matrix. Random volatility is assumed to be:


$$ln\sigma_{i,t}^{\text{2}}=\mu_{i}+\Phi_{i}\left(ln\sigma_{i,t-\text{1}}^{\text{2}}-\mu_{i}\right)+\varepsilon_{i,t},\;\varepsilon_{i,t}\sim N\left(\text{0},\omega_{i}^{\text{2}}\right)\;$$ (6)

The parameters $\mu_{i},\Phi_{i},\sigma_{i}^{\text{2}}$ are long-term fluctuation level, fluctuation persistence and fluctuation impact intensity, respectively.

Finally, by integrating time-varying parameters and stochastic volatility, the TVP-SV-VAR model can be expressed as:


$$y_{t}=X_{t}\beta_{t}+{L_{t}\varepsilon }_{t},\;\varepsilon_{t}\sim \;N(\text{0},D)$$ (7)

### 3.2. Variable description and sample selection

Bond market opening (Bo). Currently, academic circles mainly classify the measurement methods of bond market opening into two categories: measurement based on laws and regulations and measurement based on facts. The measurement based on facts can dynamically reflect trends in foreign capital flows, adjustments in risk preferences, and shocks from unexpected events, thereby enabling a more accurate assessment of the degree of bond market opening. Therefore, this paper selects the ratio of debt securities under China’s foreign financial liabilities project to GDP as the indicator to measure the degree of bond market opening. Data are sourced from the Wind Database.

Monetary policy (MP). In academic circles, the measurement indicators for monetary policy have shifted from traditional quantitative indicator methods to high-frequency identification methods. Through high-frequency changes in data such as interest rate derivative pricing and government bond term premium, as well as the futures attribute of such data, this method infers the implicit monetary policy path in real time and reflects dynamic market information and future expectations. Therefore, this paper selects the interest rate swap contract FR007 (one year) — the largest in terms of domestic interest rate derivatives trading volume — as the proxy variable for monetary policy. Data are sourced from the Wind Database.

Systemic financial risks (SFR). Currently, academic circles mainly adopt two approaches to measure systemic financial risks: one is the comprehensive index method, and the other is constructing models to measure the financial risk of individual institutions and finally synthesizing the overall systemic financial risks. The IMF suggests that developing countries, which have shorter financial market development histories and face greater difficulties in data acquisition, are more appropriate to use the comprehensive index method to measure systemic financial risks; referring to Tao and Zhu [32], this paper adopts the comprehensive index method to construct China’s systemic financial risks indicator. In view of the core defects of the traditional weighting method of the comprehensive index method in the face of complex financial systems and multi-dimensional risk monitoring — such as the static solidification of the weighting mechanism and interference from subjective factors — this paper innovatively uses the MWA-CRITIC weighting model, which realizes the adaptive adjustment of indicator weights by real-time capturing the dynamic interaction relationships among various risk dimensions, effectively overcomes the limitations of traditional static weighting models, and provides innovative technical support for the accurate identification and dynamic monitoring of systemic financial risks.

According to the actual situation of China’s economic operation and the availability of data, this paper selects six dimensions of financial institutions, the real estate market, the stock market, the money market, the foreign exchange market and the bond market, a total of 20 basic indicator pools (see Table 1). Considering that the symbolic event of the official opening of China’s bond market occurred at the end of 2010, this study selected the time interval from January 2011 to June 2024, and the frequency was monthly. All data are from the Wind database.

**Table 1:** Indicator system of each dimension.

Dimension	Indicator name	Indicator meaning and nature
Real estate market	Year-on-year growth rate of completed investment in real estate development	Reflecting the prosperity level of the real estate market (-)
	Year-on-year growth rate of commercial housing sales value	
	National housing prosperity index	
	Price to earnings ratio of the real estate industry	Reflecting the market’s expectations and confidence in the real estate industry (+)
financial institution	Deposit loan ratio	Reflecting the ability of banking and financial institutions to resist risks (-)
	Ratio of medium and long-term loans to total loans	Reflecting the debt structure of banking and financial institutions (+)
	Average price-to-earnings ratio of financial institutions	Reflecting the market’s expectations and confidence in the financial industry (+)
		Total market value of listed financial institutions
Stock market	WIND full A-share transaction amount	Reflecting the prosperity level of the stock market (+)
	WIND full A-share index	
	Average Volatility of the Shanghai Composite Index	Reflecting stock market risks (+)
Money market	Weighted interest rate of interbank pledged repo (1–7 days)	Reflecting the level of interest rate spread between long-term and short-term borrowing of funds (+)
	Fixing rate of interbank pledged repo (1-Day)	Reflecting the supply and demand of short-term funds (-)
	M2 year-on-year growth rate - M1 year-on-year growth rate	Reflecting the supply and demand situation of investment in the real economy (+)
Bond market	Yield to maturity of treasury bond (10−1 years)	Reflecting the spread between long-term and short-term assets (+)
	Yield to maturity of corporate bonds (AAA): 1 year – yield to maturity of treasury bond: 1 year	Reflecting the credit spread of different bond varieties (+)
	China: treasury bond yield to maturity: 1 year – US: treasury bond yield to maturity: 1 year	Reflecting sovereign bond spreads (+)
Foreign exchange market	Real Effective Exchange Rate Index of RMB	Reflecting the RMB exchange rate price (-)
	Year-on-year growth rate of total import and export value	Reflecting the level of economic prosperity (+)
	Foreign exchange reserve	Reflecting the ability to withstand exchange rate shocks (-)

Standardize all variables to eliminate the influence of dimensional differences. Equations (8) and (9) are the standardization processes for positive and negative indicators, respectively.


$$Z_{t}=\frac{x_{t}-min\left(x_{t}\right)}{max\left(x_{t}\right)-min\left(x_{t}\right)}$$ (8)

$$Z_{t}=\frac{{max\left(x_{t}\right)-x}_{t}}{max\left(x_{t}\right)-min\left(x_{t}\right)}$$ (9)

To construct the Systemic financial risks Index (SFR), follow these steps: First, use the MWA-CRITIC weighting model to dynamically assign weights to indicators within each financial subsystem and synthesize the risk indices for each subsystem. Second, based on the financial risk indices of the six subsystems, continue using the MWA-CRITIC weighting model to determine the weights among the subsystems and synthesize the comprehensive systemic financial risks index (SFR) for China. The mathematical expression of the traditional static CRITIC weighting method is as follows:


$$\begin{array}{c} C_{j}=\sigma_{j}\sum {\mathrm{\backslash nolimits}}_{i=\text{1}}^{n}\left(\text{1}-r_{ij}\right),j=\text{1},\text{2},\ldots ,n \end{array}$$ (10)

$$W_{j}=\frac{C_{j}}{\sum_{j=\text{1}}^{n}C_{j}},\;j=\text{1},\text{2},\ldots ,n$$ (11)

In the formula, $C_{j}\;$represents the amount of information contained in the *j*th financial subsystem, $\sigma_{j}\;$is the standard deviation of the *j*th financial subsystem, $r_{ij}$ is the correlation coefficient between the *i*th and *j*th financial subsystems, and $W_{j}$ is the static weight of the *j*th financial subsystem.

To accurately reflect the time-varying correlation characteristics of various financial subsystems, we introduce the Moving Weighted Average (MWA) method to calculate the $\sigma_{j}$ standard deviation between indicators within financial subsystems and between financial subsystems. The mathematical expression is as follows:


$$\sigma_{jt}=\sqrt{\frac{\text{1}}{N-\text{1}}\sum {\mathrm{\backslash nolimits}}_{k=t-N+\text{1}}^{t}{(x_{jk}-\mu_{jt})}^{\text{2}}},\;\mu_{jt}=\frac{\text{1}}{N}\sum {\mathrm{\backslash nolimits}}_{k=t-N+\text{1}}^{t}x_{jk}\;$$ (12)

$$\begin{array}{c} {\sigma_{j}}^{MA}=\frac{\text{1}}{N}\sum {\mathrm{\backslash nolimits}}_{k=t-N+\text{1}}^{t}\sigma_{jk} \end{array}$$ (13)

$${C_{j}}^{MA}={\sigma_{j}}^{MA}\sum {\mathrm{\backslash nolimits}}_{i=\text{1}}^{n}\left(\text{1}-r_{ij}\right),\;j=\text{1},\text{2},\ldots ,n$$ (14)

$${W_{j}}^{MA}=\frac{{C_{j}}^{MA}}{\sum_{j=\text{1}}^{n}{C_{j}}^{MA}},\;j=\text{1},\text{2},\ldots ,n$$ (15)

In the formula, the window length *N* is set to 5 days, where $\mu_{jt}$ represents the mean of financial subsystem *j* at time *t*. Substituting *t*he obtained time-varying standard deviation into equation (3) allows us to derive the time-varying weights ${W_{j}}^{MA}$ for each financial subsystem. After determining the weights for each indicator, we can calculate the financial risk index *SFRX*_*j*_ for each subsystem and the systemic financial risks index (*SFR*) using the following forms. The formulas are as follows:


$$\begin{array}{c} {SFRX}_{j,t}=\sum {\mathrm{\backslash nolimits}}_{i=\text{1}}^{m}W_{j,it}*X_{j,it},i=\text{1},\text{2},\ldots ,m;j=\text{1},\ldots ,\text{6} \end{array}$$ (16)

$$\begin{array}{c} {SFR}_{t}=\sum {\mathrm{\backslash nolimits}}_{i=\text{1}}^{n}W_{jt}*{SFRX}_{j,t},j=\text{1},\ldots ,\text{6} \end{array}$$ (17)

**Fig 1:**
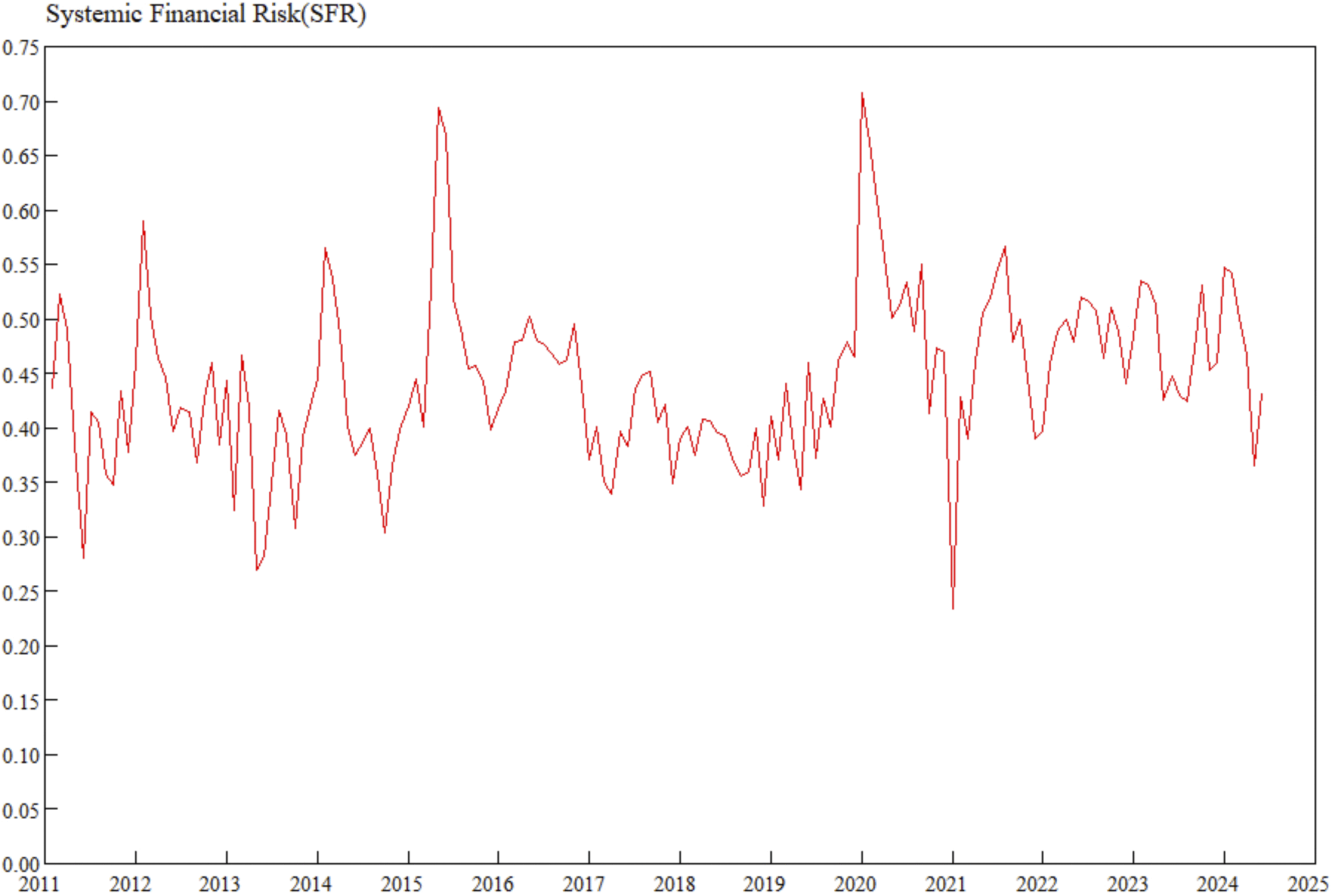
Systemic financial risks index.

In the formula, ${SFRX}_{j,t}$ represents the financial risk index of subsystem *j* in the financial system at time *t*, $W_{j,it}$ denotes the weight of *t*he *i*th indicator of subsystem *j* at time *t*,$\;X_{j,it}$ is the value of the *i*th indicator of subsystem *j* at time *t*,$\;W_{jt}$ represents the weight of the financial stress index for the *j*th financial subsystem at time *t*, and ${SFR}_{t}$ is *t*he comprehensive index of systemic financial risks at time *t*. The synthesized systemic financial risks index is shown in Fig 1.

As shown in Fig 1, the systemic financial risks index constructed in this study has a similar trend to that of Liang [33] at the same time points, realizing mutual verification. In two extreme scenarios — the domestic stock market volatility in 2015 and the COVID-19 pandemic shock in 2020 — the dynamic weighting mechanism of the MWA-CRITIC framework more accurately captures the risk accumulation process under major emergencies compared with traditional static weighting, correcting the underestimation bias of static weights for extreme risks and providing forward-looking support for countercyclical regulation. In stages: From January 2011 to September 2014, despite external shocks from the European debt crisis, China’s timely intervention policies controlled risk volatility, maintaining an overall safe state; after the central bank cut interest rates in November 2014, the A-share market experienced irrational surges within 6 months, leading to rapid risk accumulation; the stock market adjustment in mid-2015 triggered a liquidity crisis, and risks were concentratedly released through market clearing, returning to a reasonable range by the end of the year. After the “August 11 exchange rate reform” in 2015, exchange rate volatility expanded and uncertainty in cross-border capital flows increased, expanding risk exposure; in 2016, the regulatory authorities introduced policies such as reserve requirement ratio cuts, interest rate cuts, and margin trading adjustments, with effects becoming apparent after October. From the end of 2019 to the beginning of 2020, the COVID-19 pandemic shocked the global economy — China’s GDP growth slowed down, the real estate industry was severely hit, and market sentiment fell to a historical low; the Party Central Committee and local governments launched emergency responses and introduced a policy package including “six stabilities” and “six guarantees”. From the first quarter of 2021, economic growth turned positive, and risks remained controllable until June 2024, verifying the institutional resilience of China’s risk prevention and control system.

## 4 Empirical results

### 4.1. Descriptive statistics

Table 2 presents the statistical characteristics of the Bond market opening (BO), Monetary Policy (MP), and Systemic financial risks Index (SFR), listing the descriptive statistics results of the three variables. As shown in the table, during the period 2011–2024, the mean value of Bond market opening (BO) is 2.8664, with a minimum value of 1.4229, a maximum value of 5.1815, and a standard deviation of 0.6974. A relatively high mean value, combined with a relatively large standard deviation and a wide range between the minimum and maximum values, reflects that the process of bond market opening may have been affected by various internal and external factors during this period, and the market structure and development pace have undergone relatively drastic changes. Compared with the bond market opening index, the mean values, standard deviations, and ranges of the Monetary Policy Index and Systemic financial risks Index are all at relatively lower levels. This indicates that these two indicators may have been affected by relatively stable policy regulation and risk control during the period, with no significant fluctuations in their values, which to a certain extent reflects the stability of the financial system in these two aspects.

**Table 2:** Summary of statistics for BO, MP, and SFR.

*Indicator Name*	*N*	*Mean*	*Std. Dev*	*Min*	*Max*
BO	162	2.8664	0.6974	1.4229	5.1815
MP	162	0.0246	0.0135	0.0030	0.0463
SFR	162	0.4430	0.0762	0.2343	0.7077

### 4.2. Data stationarity test and model parameter estimation

From Table 3 of the unit root test, it can be seen that the p-values of the three original data series are all significant after first-order differencing, indicating they can be treated as stationary time series for subsequent empirical analysis. Based on the information criteria in Table 4 and considerations of model stability, the TVP-SV-VAR model in this study selects a 1st-order lag and sets 10,000 simulation runs for the posterior parameters. The parameter estimation results are shown in Table 5. The results indicate that, at the 1% significance level, the Geweke Test results are unable to reject the null hypothesis that the estimated parameters converge to the posterior standard distribution, suggesting that during the iterative cycle, the burn-in period is already sufficient to effectively bring the Markov chain to concentration. Additionally, the ineffective influence factors for the parameters are generally low (with a maximum of only 73.14), indicating that the effective samples obtained from the model are adequate for estimation, meeting the requirements of posterior statistical inference, and showing that the posterior mean is close to the true parameter values. This demonstrates that the model parameter simulation results are very effective. Based on the above results, both the parameter and model estimates are valid, and subsequent time-varying impulse response function analysis can be conducted.

**Table 3:** ADF test table.

*Variable*	*Difference order*	*t*	*p*	*Critical value*		
				1%	5%	10%
BO	0	−3.580	0.034	−3.996	−3.428	−3.138
	1	−12.272	0.000	−3.996	−3.429	−3.138
	2	−11.525	0.000	−3.997	−3.429	−3.138
SFR	0	−7.312	0.000	−3.996	−3.428	−3.137
	1	−11.303	0.000	−3.996	−3.429	−3.138
	2	−10.990	0.000	−3.997	−3.429	−3.138
MP	0	−2.247	0.461	−3.996	−3.428	−3.137
	1	−16.700	0.000	−3.996	−3.428	−3.138
	2	−10.475	0.000	−3.998	−3.429	−3.138

*P < 0.05、**P < 0.01、***P < 0.001

**Table 4:** Lag order information criteria summary.

*Lag*	*Log L*	*LR*	*FPE*	AIC	SC	HQ
0	355.0052	NA	1.12e-05	−2.885	−2.842	−2.868
1	1153.375	1570.564*	1.74e-08*	−9.356*	−9.184*	−9.286*
2	1159.755	12.393	1.77e-08	−9.334	−9.033	−9.213
3	1167.870	15.564	1.79e-08	−9.327	−8.897	−9.154

* indicates lag order selected by the criterion.

**Table 5:** Results and diagnostics of parameter estimation.

*Variable*	*Mean*	*Standard deviation*	*95% confidence interval*	*Geweke test*	*Invalid impact factor*
sb1	0.0224	0.0025	[0.0182-0.0278]	0.569	11.37
sb2	0.0228	0.0026	[0.0184-0.0286]	0.658	17.75
sa1	0.0319	0.0047	[0.0244-0.0427]	0.945	26.43
sa2	0.0639	0.0169	[0.0403-0.1045]	0.111	40.75
sh1	0.5661	0.1122	[0.3674-0.7999]	0.854	53.73
sh2	0.6668	0.0988	[0.5017-0.8887]	0.047	73.14

### 4.3. Time-varying stochastic volatility analysis

**Fig 2:**
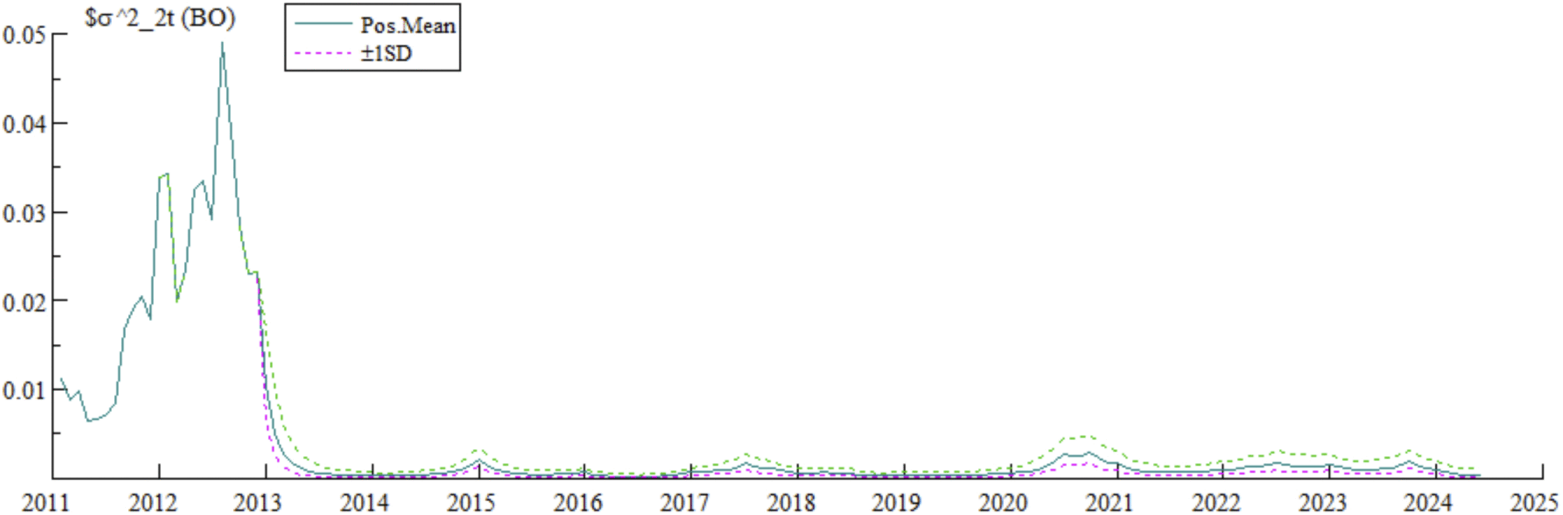
Stochastic volatility characteristics of the open bond market.

As shown in Fig 2, the stochastic volatility of bond market opening (BO) was relatively high before 2014, then decreased to near zero and remained stable, with only slight fluctuations in 2015, 2017, 2020, and 2022. The specific driving factors were as follows: In August 2010, the People’s Bank of China issued a notice on the pilot program for overseas institutions to invest in the interbank bond market, marking the official launch of bond market opening and the initiation of the “Global Connect” model. However, initial cross-border capital inflows amplified instability during the opening process, pushing up volatility. Subsequent steady progress in opening stabilized volatility: In November 2014, an ETF tracking the China High-Quality Bond Index was listed on the New York Stock Exchange, enhancing international market recognition; in July 2017, the “Bond Connect” was launched, supplementing the “Global Connect” to expand access channels for foreign investors and upgrading opening; in April 2020, RMB-denominated Chinese government bonds and policy bank bonds were included in the Bloomberg Barclays Global Aggregate Index, marking the entry of Chinese bond assets into the benchmark allocation pool of global institutional investors, reflecting international recognition of opening achievements; in November 2020, the Measures for the Administration of Domestic Securities and Futures Investments by Qualified Foreign Institutional Investors and RMB Qualified Foreign Institutional Investors was issued; in May 2023, the “Swap Connect” was launched, allowing overseas institutions to participate in domestic Treasury bond futures, interest rate swaps (IRS), and other risk management tools to hedge duration and exchange rate risks, accelerating the inflow of allocation-based foreign capital.

**Fig 3:**
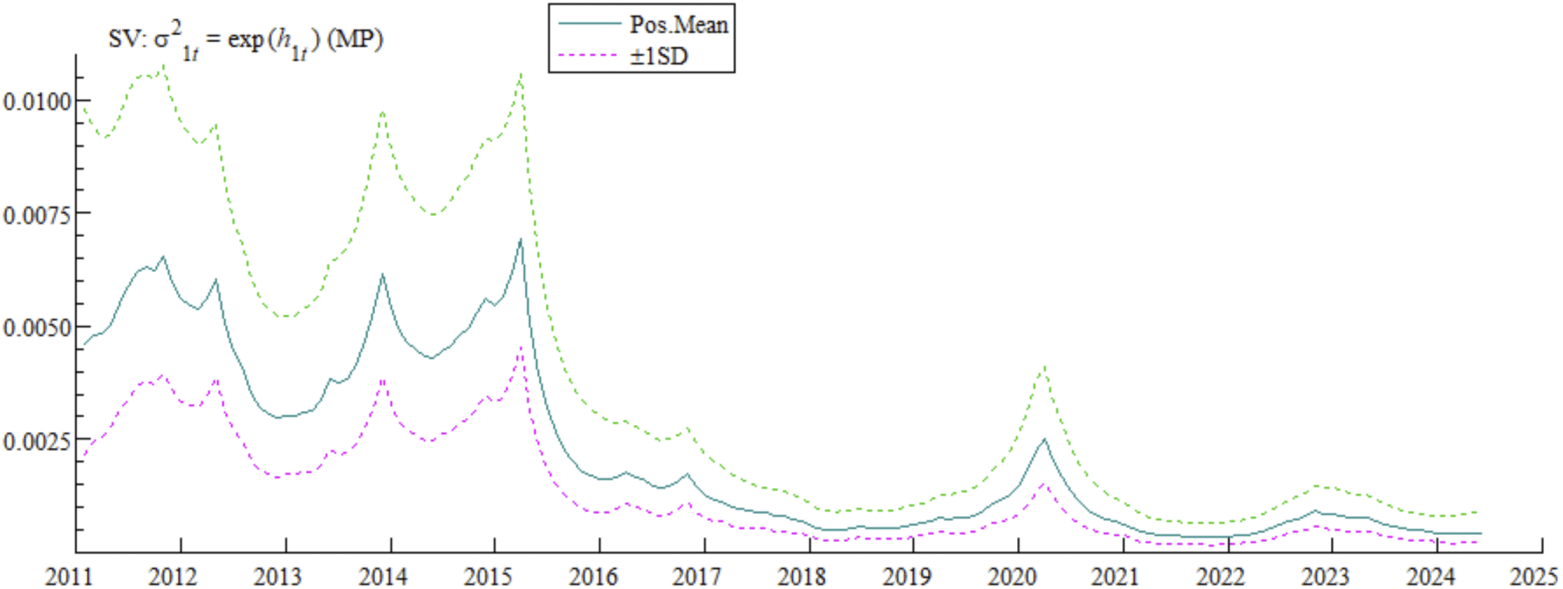
Stochastic volatility characteristics of monetary policy.

From the perspective of monetary policy stochastic volatility (see Fig 3), this indicator fluctuated in a relatively high range from 2011 to 2015, then approached zero and stabilized, with only a certain degree of volatility in 2020. Looking back to around 2011, China’s interest rate marketization reform entered a new stage of accelerated advancement, with significantly accelerated reform steps. Particularly in 2013, China fully liberalized the upper limit of deposit interest rates and established the Standing Lending Facility (SLF) and Medium-term Lending Facility (MLF), marking a key leap in interest rate marketization reform and the establishment of a modern monetary policy framework dominated by price-based tools. In October 2015, with the last adjustment of deposit and lending benchmark interest rates and the complete liberalization of requirements on the upper and lower limits of deposit interest rates, China’s interest rate control system was basically fully liberalized. In August 2019, the People’s Bank of China improved the loan prime rate (LPR) formation mechanism, promoting the “merger of two tracks into one” for lending rates, and basically formed a market-oriented interest rate formation and transmission mechanism, as well as a relatively complete market-oriented interest rate system. Against the backdrop of this series of major reforms, the stochastic volatility of monetary policy showed sharp fluctuations, fully reflecting the complexity and dynamics of the interest rate marketization reform process.

**Fig 4:**
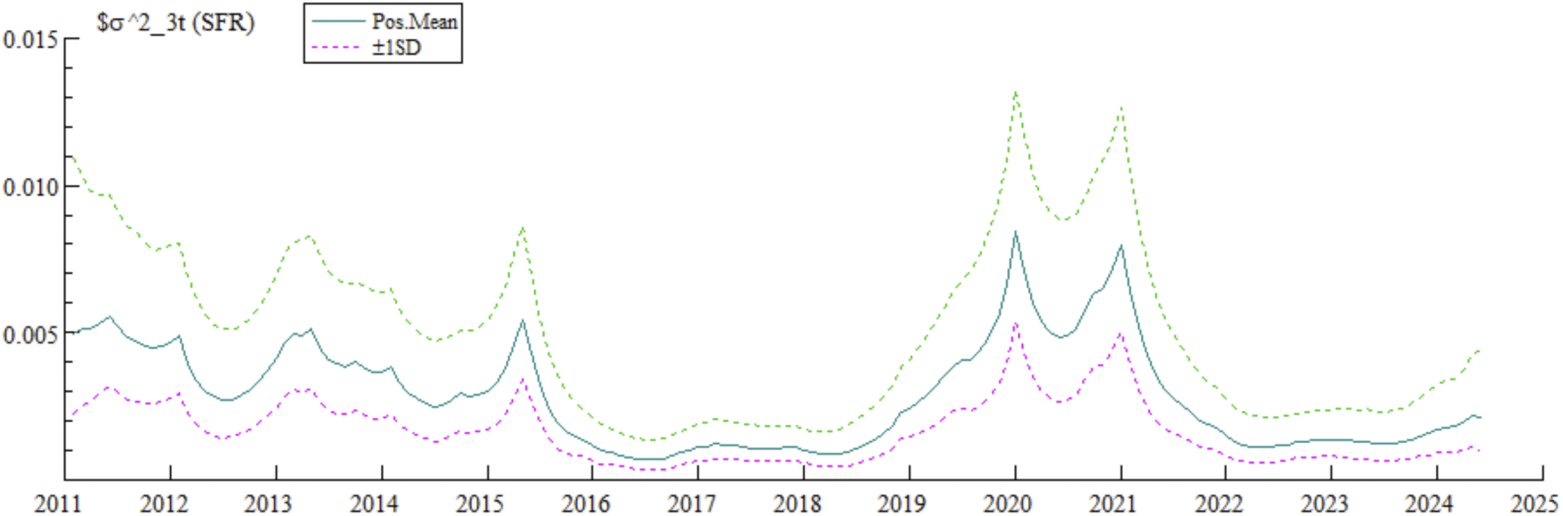
Systemic financial risks stochastic volatility characteristics.

As shown in Fig 4, the stochastic volatility of China’s systemic financial risks exhibits phased characteristics: significant fluctuations from 2011 to 2016, basically stabilizing from 2017 to 2019, and significantly increasing after 2020 due to multiple shocks. The root cause of this volatility lies in the rapid rise of China’s macro leverage ratio in the post-financial crisis era. To resolve risks, the Central Economic Work Conference at the end of 2015 listed “cutting overcapacity, reducing inventory, deleveraging, lowering costs, and strengthening weak links” as core tasks for 2016. Since then, the stochastic volatility of risks has significantly decreased and stabilized, fully reflecting the phased achievements of China’s prevention and resolution of systemic financial risks. The renewed sharp rise in risk stochastic volatility after 2020 is mainly attributed to four factors: First, the decline in potential economic growth — affected by deepening population aging and the transformation of industrial drivers, China’s economic growth rate dropped from 14.23% in 2007 to 5.95% in 2019, and the slowdown in growth exacerbated the deterioration of debt in various sectors, pushing up risks; second, short-term shocks from real estate regulation — since the beginning of 2018, the long-term mechanism of “housing is for living in, not for speculation” and “city-specific policies” has been implemented, which is conducive to deflating bubbles in the long term but led to the deterioration of debt of real estate entities in the short term, triggering liquidity risks; third, direct impact of the COVID-19 pandemic — dynamic lockdown measures led to operational and financial deterioration of small and medium-sized service enterprises, sharp declines in local government fiscal revenue, and increased debt-servicing burdens, increasing financial market fragility; fourth, persistent impact of China-U.S. economic competition — in March 2018, the U.S. government launched a trade war against China, impacting China’s foreign trade and overall economy, spreading to the financial sector, and increasing risk instability.

### 4.4. The impact of bond market opening on systemic financial risks

**Fig 5:**
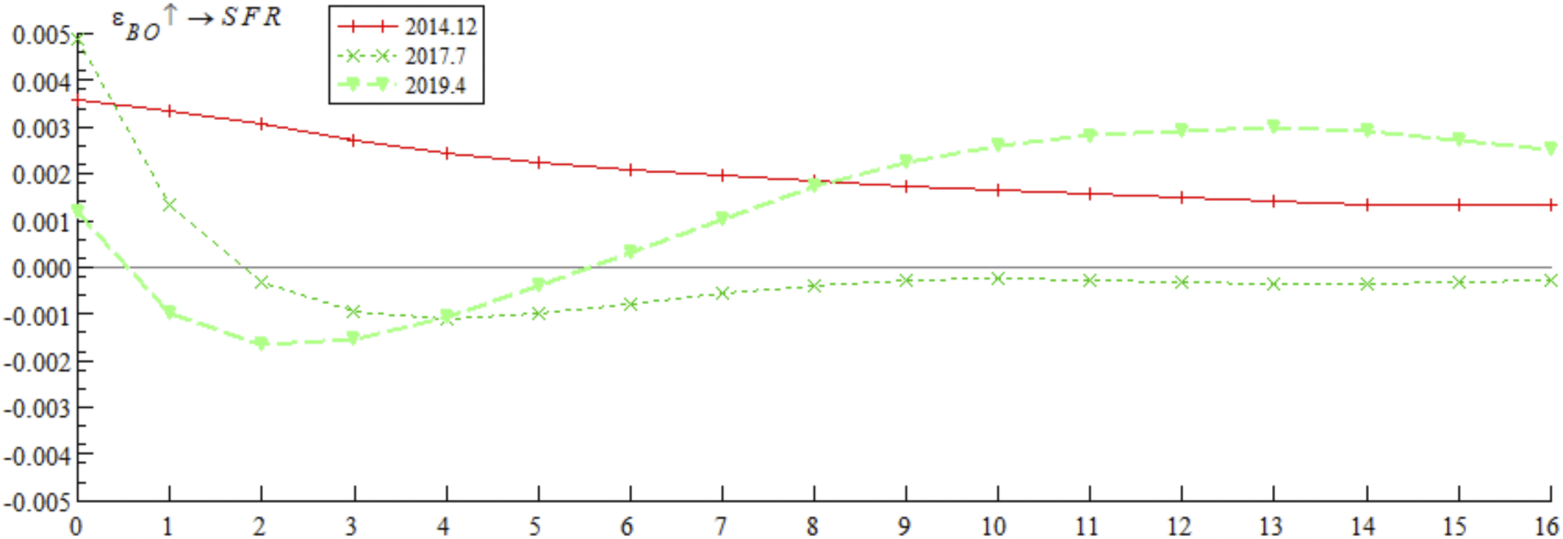
Impulse response results of bond market opening on systemic financial risks at three different time points.

Fig 5 presents the dynamic trajectory of the impulse response of systemic financial risks to bond market opening policies at three key time points: December 2014 (initial opening period — the first listing of China Bond ETF on the New York Stock Exchange), July 2017 (adjustment opening period — official launch of the “Bond Connect”), and April 2019 (deepened opening period — first inclusion of Chinese interest rate bonds in the global aggregate bond index). The empirical results show that the impulse response of systemic financial risks to bond market opening policy shocks differs significantly at different time points. The shock in December 2014 showed a positive response in both the short and long terms; the shocks in July 2017 and September 2019 showed positive responses in the short term but quickly shifted to the negative response range, with divergent trends in subsequent periods. These results indicate that the impact of bond market opening on China’s systemic financial risks exhibits time-varying characteristics, which may stem from the interaction between external event shocks and different stages of opening policy implementation.

**Fig 6:**
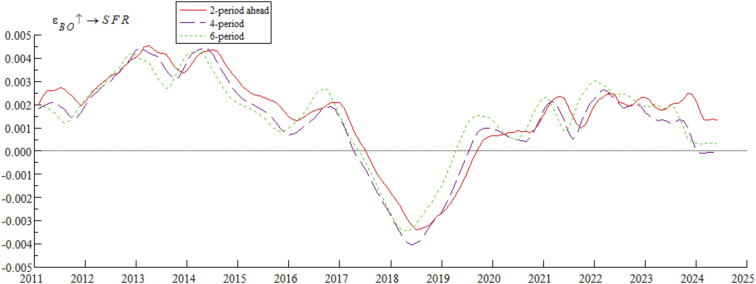
Impulse response results of bond market opening on systemic financial risks at 2, 4, and 6 periods ahead.

Fig 6 shows the impulse response curves of systemic financial risks to a one-unit positive shock of bond market opening under the conditions of 2-period, 4-period, and 6-period ahead, systematically depicting the differential impact of bond market opening on systemic financial risks in different time intervals. Specifically, from 2011 to 2016, short-term arbitrage capital flowed frequently through QFII, exacerbating fluctuations in exchange rates and foreign exchange reserves — a characteristic highly consistent with the dual crisis theory mechanism revealed by Kaminsky and Reinhart [34], i.e., unordered short-term capital flows tend to trigger market fragility. From 2017 to 2019, the “Bond Connect” attracted long-term foreign capital, and the inclusion of Chinese bonds in international authoritative indexes (along with the opening of credit rating services) significantly mitigated systemic financial risks — a phenomenon consistent with the empirical conclusion of Fratzscher [35] that “long-term cross-border capital inflows can enhance financial market stability”. After 2020, foreign bond holdings exceeded RMB 4 trillion, intensifying cross-border risk contagion; for example, the Federal Reserve’s interest rate hikes in 2022 triggered a wave of foreign selling, which further confirms the research view of Forbes and Warnock [36] that global liquidity shocks tend to trigger violent turbulence in emerging market economies’ financial markets. The empirical research of domestic scholar Yan et al. [37] also supports this law, showing that the scale of cross-border capital flows is significantly positively correlated with the level of systemic financial risks.

Based on the empirical analysis in Figs 5 and 6, it is evident that the impact of bond market opening on China’s systemic financial risks exhibits time-varying characteristics. Through mechanisms such as cross-border capital flows, cross-border risk contagion, and market structure optimization, bond market opening dynamically influences systemic financial risks by promoting, inhibiting, and then promoting it again. These findings suggest that while advancing the high-quality development of the bond market, Chinese regulatory authorities urgently need to improve monitoring, evaluation, and early warning mechanisms for cross-border capital flows, optimize the bond market credit rating system, enhance the variety of bonds and their derivatives, and strengthen international financial regulatory collaboration. These measures are essential to prevent the occurrence of domestic systemic financial risks.

### 4.5. The impact of monetary policy on systemic financial risks

**Fig 7:**
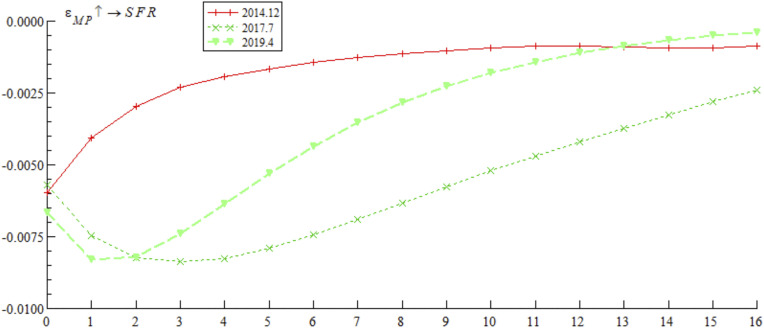
Impulse response results of monetary policy on systemic financial risks at three different time points.

Fig 7 reports the impulse response curves of monetary policy on systemic financial risks at three key time points of bond market opening policy: December 2014, July 2017, and April 2019. The empirical results show that: (1) The initial values of impulse responses at all-time points are similar, and the impulse curves are overall in the negative range, indicating that tight monetary policy promptly and effectively curbs systemic financial risks — consistent with the core idea of the Taylor rule proposed, which states that interest rate tools in monetary policy can effectively stabilize financial market fluctuations and maintain market stability. (2) Interest rate marketization reform promotes the differentiation of mitigation effects. The impulse curve in December 2014 shows that the mitigation effect of tight monetary policy on systemic financial risks decays rapidly within the first 3 periods; in contrast, the impulse curves in July 2017 and April 2019 show that the mitigation effect continues to strengthen in the early stage, with a significantly slower decay rate in the middle and late stages. The core reason for this difference lies in the implementation of the Macro Prudential Assessment (MPA) system in 2017 and the Loan Prime Rate (LPR) mechanism in 2019, which effectively unblocked the monetary policy transmission channel and significantly enhanced the sustainability of its role in curbing systemic financial risks. Woodford [38] also pointed out that building a sound policy transmission mechanism is the core prerequisite for ensuring the sustained effectiveness of monetary policy; the empirical research of domestic scholar Ma et al. [39] based on China’s financial market practice further confirms that the Macro Prudential Assessment (MPA) system can significantly enhance the sustainability of monetary policy regulation effects by constraining the operating behavior of financial institutions.

**Fig 8:**
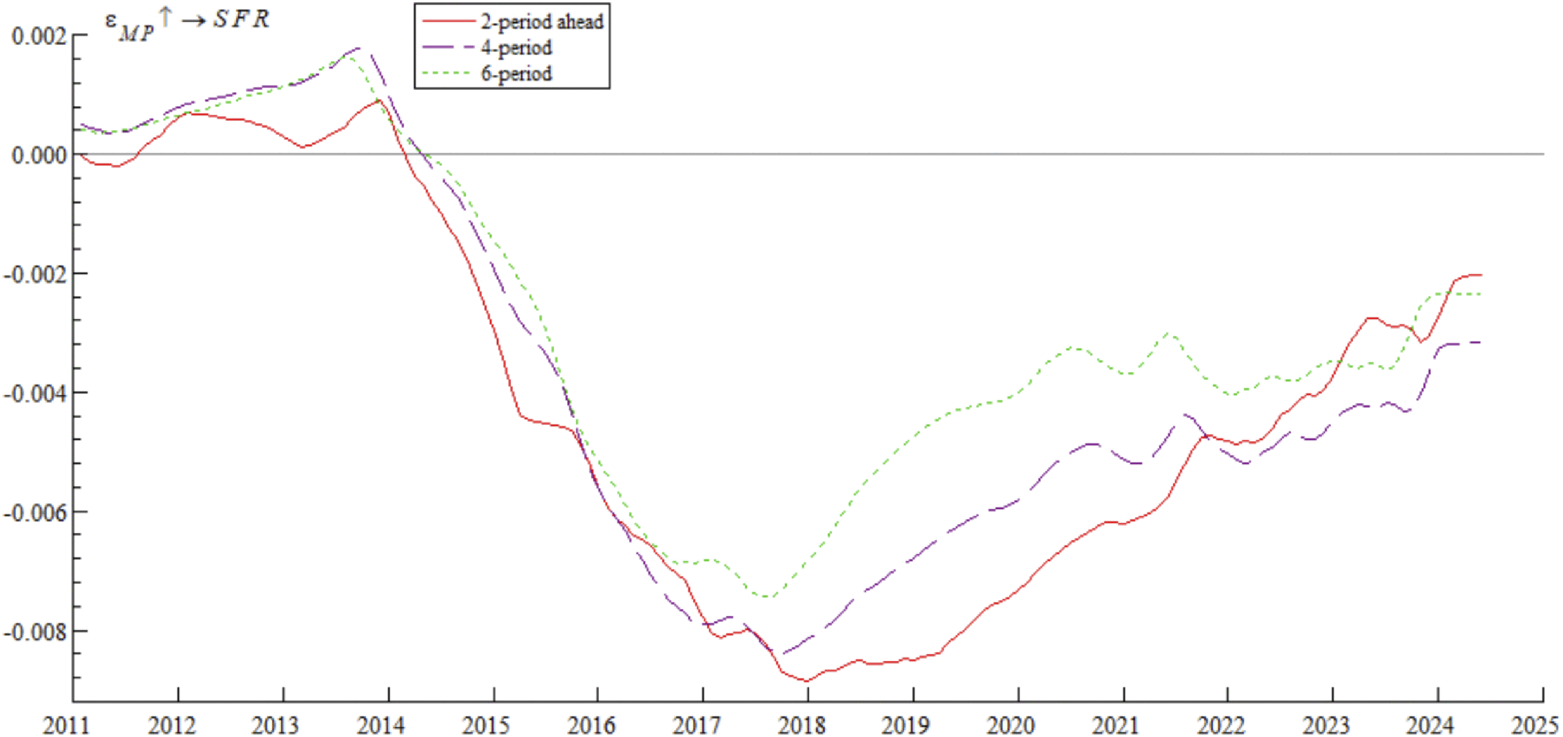
Impulse response results of monetary policy on systemic financial risks at 2, 4, and 6 periods ahead.

Fig 8 shows the impulse response curves of systemic financial risks to a one-unit positive shock of monetary policy under the conditions of 2-period, 4-period, and 6-period ahead, dynamically analyzing the differential impact of monetary policy on systemic financial risks in different time intervals. Specifically, after the establishment of the market interest rate pricing self-discipline mechanism in 2013, the policy response shifted from near zero to the negative range, confirming the research conclusion of McCallum [40] that optimizing the market interest rate transmission mechanism improves the effectiveness of monetary policy. In 2017, the MPA curbed systemic financial risks by constraining the capital adequacy ratio of financial institutions — consistent with the view of Borio [41] that macro-prudential supervision strengthens risk resistance. The 2019 Loan Prime Rate (LPR) reform pushed the mitigating effect to its peak, but after 2020, the effect was weakened by the impact of the pandemic and the Federal Reserve’s interest rate hikes. This is precisely the practical manifestation of the Impossible Trinity theory proposed by Obstfeld and Rogoff [42], that is, the independence of monetary policy is subject to external constraints under open conditions. Domestic research also provides evidence for this: Chen et al. [43] empirically show that the LPR reform significantly improves the transmission efficiency of monetary policy, but frequent cross-border capital flows may weaken this policy effect to a certain extent.

The empirical study in Figs 7 and 8 shows that contractionary monetary policy inhibits systemic financial risks through mechanisms such as interest rate transmission, liquidity regulation, and financial institution behavior control. However, this inhibitory effect gradually weakens under external structural shocks. These findings suggest that Chinese regulatory authorities should continue to deepen interest rate marketization reforms, promote the improvement of macro-prudential policy frameworks, and establish a macro-prudential management framework for cross-border capital flows. By enhancing policy transmission efficiency and strengthening regulatory synergy, they can effectively enhance the role of monetary policy in controlling systemic financial risks.

### 4.6. Analysis of the interaction effect between bond market opening and monetary policy

**Fig 9:**
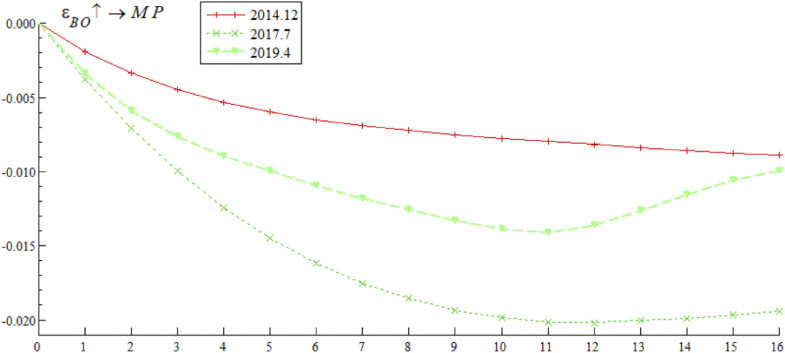
Impulse response results of monetary policy on bond market opening at three different time points.

Fig 9 shows that at three different stages of bond market opening (December 2014, July 2017, and April 2019), the impulse response curves of a one-unit positive shock of bond market opening on monetary policy continue to decline in the negative range, indicating that bond market opening weakens the effectiveness of monetary policy. The empirical research of Hu et al. [44] also draws a similar conclusion, noting that cross-border capital flows driven by China-U.S. interest rate spreads significantly reduce the autonomy of China’s monetary policy. It is worth noting that the impulse response value declined most significantly in July 2017, followed by April 2019, and relatively weakly in December 2014 — reflecting that the mitigating effect of bond market opening on monetary policy exhibits dynamic adjustment characteristics, which may be deeply related to adjustments in domestic interest rate marketization policies and cross-border capital volatility caused by domestic and foreign financial market turbulence.

**Fig 10:**
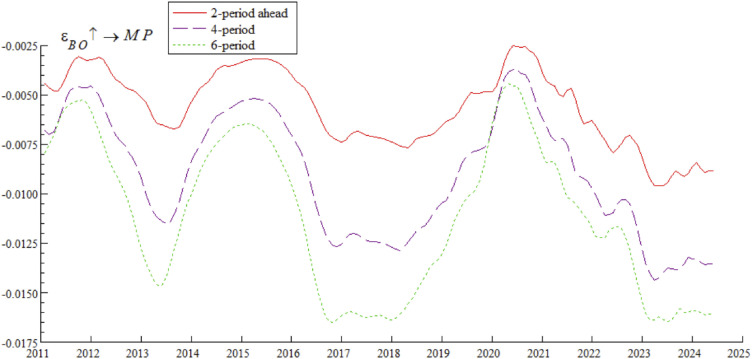
Impulse response results of monetary policy under bond market opening at 2, 4, and 6 periods ahead.

Fig 10 shows the dynamic process of the impact of bond market opening on monetary policy. Under the conditions of 2-period, 4-period, and 6-period ahead, the impulse response curves all show a gradual wave-like downward trend, indicating that the impact of bond market opening on monetary policy exhibits time-varying characteristics. Specifically, after the breakthrough in interest rate marketization in 2013, the regulatory effect of traditional monetary policy tools weakened — consistent with Friedman’s [45] judgment that “policy tools need to be transformed in the process of marketization”. The narrowing of China-U.S. interest rate spreads in 2018 triggered capital outflows, forcing the central bank to balance monetary policy between stabilizing growth and stabilizing the exchange rate — a phenomenon that confirms the view of Calvo and Mishkin [46] that “emerging market policies are vulnerable to external shocks”. The interest rate spread inversion in 2022 exacerbated constraints on monetary policy — consistent with the conclusion of Jiang et al. [47] that cross-border capital flows disrupt monetary policy transmission.

**Fig 11:**
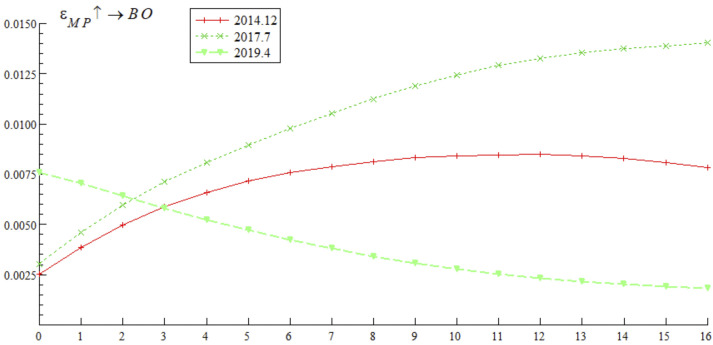
Impulse response results of monetary policy on bond market opening at three different time points.

Fig 11 focuses on three key time points of bond market opening (December 2014, July 2017, and April 2019), presenting the impulse response characteristics of a one-unit positive shock of monetary policy on bond market opening. In comparison, during the interest rate marketization reform promotion stage (2013–2017), the promoting effect of monetary policy on bond market opening gradually strengthened, reaching its peak during the LPR reform in 2019, but then the promoting effect decayed rapidly and fell below the previous high — reflecting that the impact of monetary policy on bond market opening is undergoing structural reshaping. On the one hand, although interest rate marketization reform improves the regulatory effect by optimizing the monetary policy framework, the increased market interest rate volatility caused by deepened reform challenges the sustainability of the policy regulatory effect. Bayoumi et al. [48] pointed out that increased market interest rate volatility in the late stage of interest rate marketization increases the difficulty of monetary policy operations and weakens the sustained promoting effect of policies on market opening. On the other hand, against the backdrop of exchange rate marketization reform, cross-border capital flows continue to disrupt the independence and regulatory effect of domestic monetary policy, forming a certain degree of policy effect offset. The empirical research of domestic scholar Qian et al. [49] shows that although interest rate marketization reform optimizes the monetary policy framework, the arbitrage behavior of cross-border capital significantly weakens the regulatory effect of policies on bond market opening.

**Fig 12:**
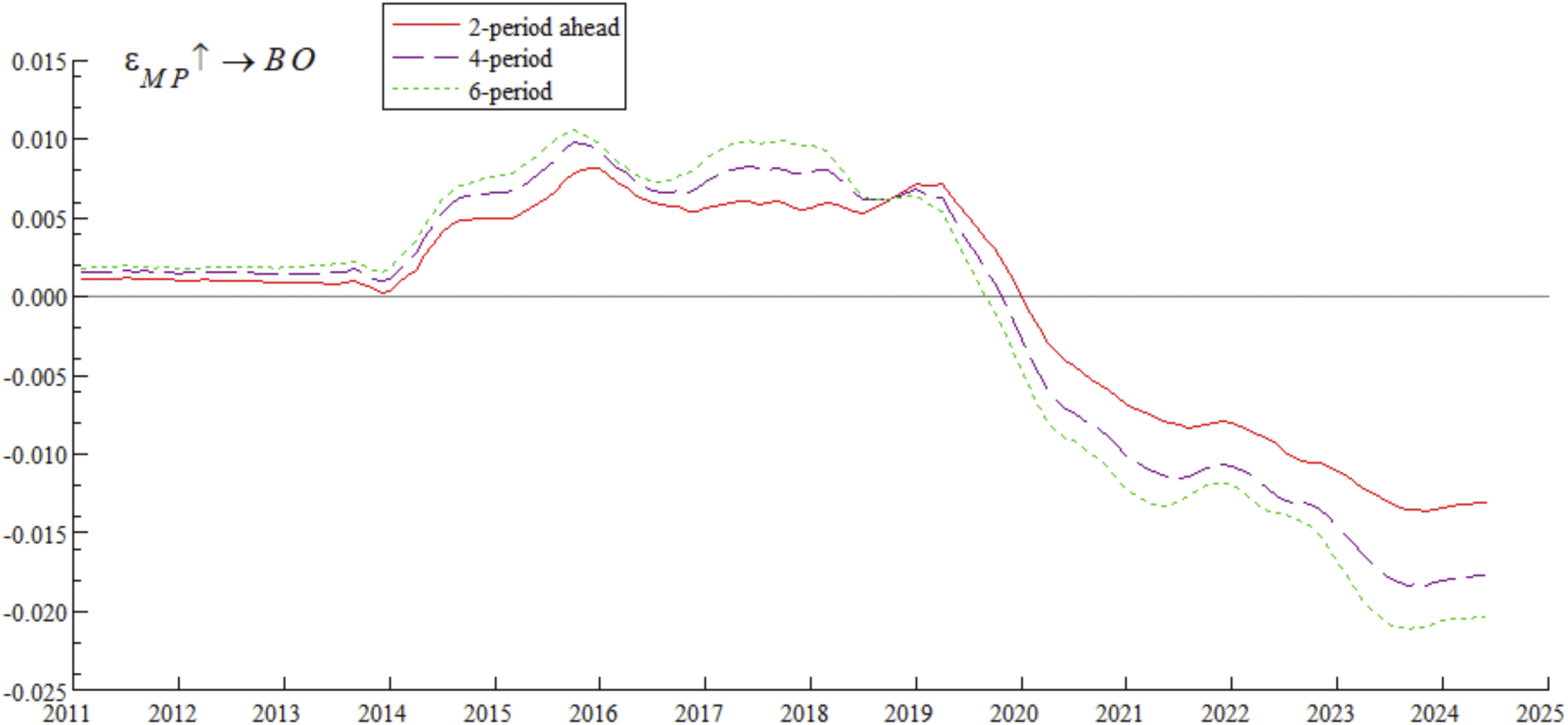
Impulse response results of monetary policy on bond market opening at 2, 4, and 6 periods ahead.

Fig 12 shows that under the conditions of 2-period, 4-period, and 6-period ahead, the trends of the impulse response curves of a one-unit positive shock of monetary policy on bond market opening are basically consistent but exhibit significant time-varying characteristics. The differences mainly stem from the interaction of interest rate marketization reform, adjustments to bond market opening policies, and intensified cross-border capital volatility. Specifically, from 2011 to 2014, the inefficient transmission of the central bank’s monetary policy, combined with the slow inflow of foreign capital under the QFII approval system, led to insufficient participation of foreign capital in the bond market, resulting in a weak impact of monetary policy on bond market opening; from 2015 to 2018, the basic liberalization of interest rate controls and the implementation of the “Bond Connect” formed a policy synergy, and the advantage of China-U.S. interest rate spreads attracted foreign capital inflows, promoting the growth of foreign bond holdings — consistent with the theoretical conclusion of Dornbusch [50] that interest rate spreads drive cross-border capital flows; after 2019, under the influence of China-U.S. interest rate spreads and cross-border exchange arbitrage, the regulatory role of monetary policy on bond market opening underwent structural reversal. The expanded China-U.S. interest rate spreads in 2019–2020 weakened the mitigating effect of loose policies; the interest rate spread inversion in 2022 triggered foreign selling, forming negative feedback in policy interest rate transmission; the USDCNY swap point entered a negative range in 2023, spurring exchange arbitrage and further weakening the monetary policy effect. This series of changes confirms the extended view of Eichengreen et al. [51] based on the “original sin theory” of emerging economies — that emerging economies are vulnerable to dual shocks from interest rate spreads and exchange rate volatility — and is also consistent with the empirical conclusion of domestic scholar Yan et al. [52] that “cross-border capital flows disrupt the implementation effect of monetary policy”.

Based on the analysis of Figs 9, 10, 11 and 12, it is evident that monetary policy and bond market opening exhibit a significant interconnection effect, which is primarily constrained by the progress of domestic institutional reforms and fluctuations in cross-border capital flows. Specifically, bond market opening imposes constraints on the autonomy and effectiveness of monetary policy through domestic and foreign interest rate linkages and the interest rate parity mechanism. These constraints manifest cyclical fluctuations in response to changes in the macroeconomic and financial environment, as well as policy frameworks.

Concurrently, monetary policy exerts a positive promotional effect on the development of bond market opening via interest rate transmission mechanisms and market expectation management. However, this effect has been completely offset and reversed since 2019 due to cross-border capital fluctuations.

## 5. Robustness tests

### 5.1 Robustness test of bond market opening

**Fig 13:**
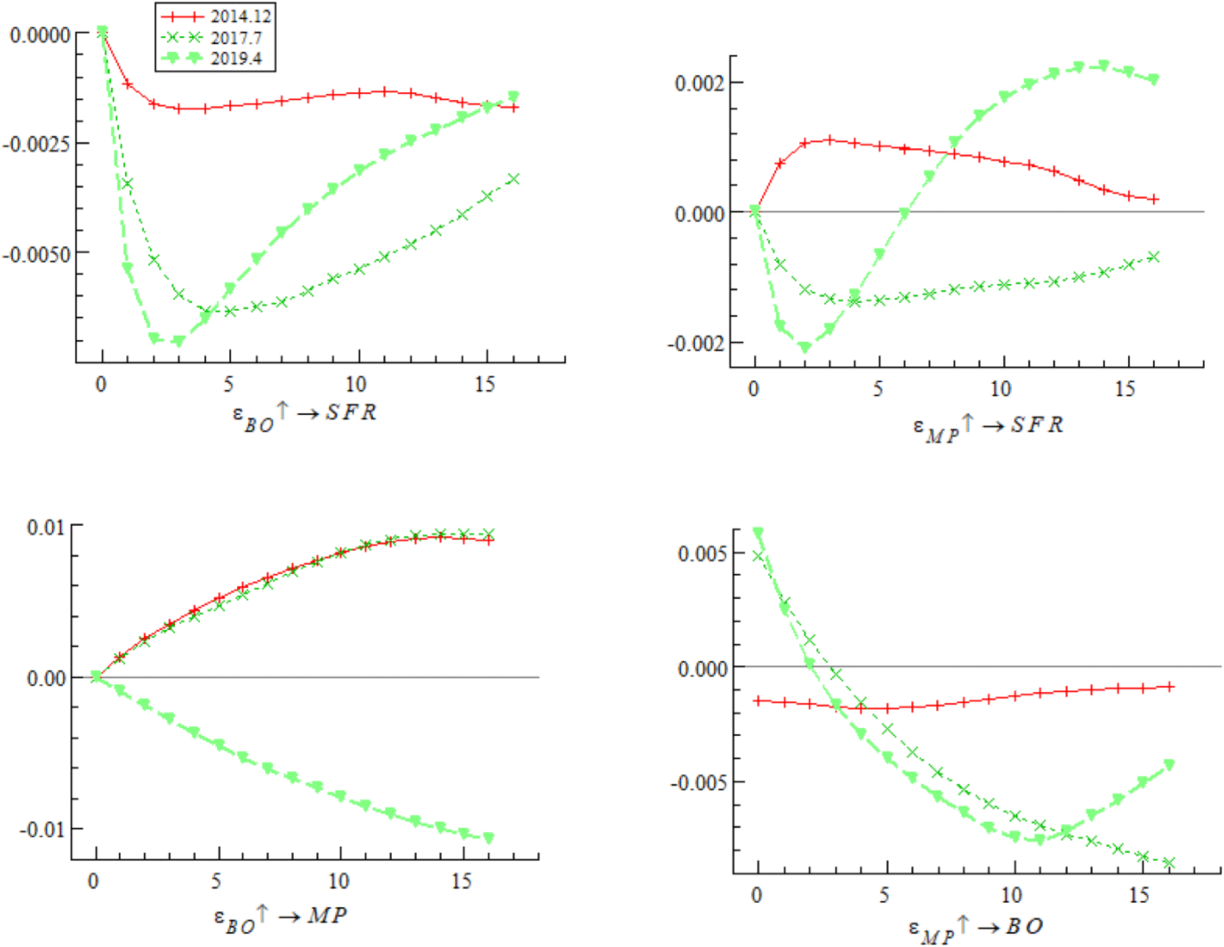
Robustness test of impulse responses at different ahead periods (Bond market opening).

**Fig 14:**
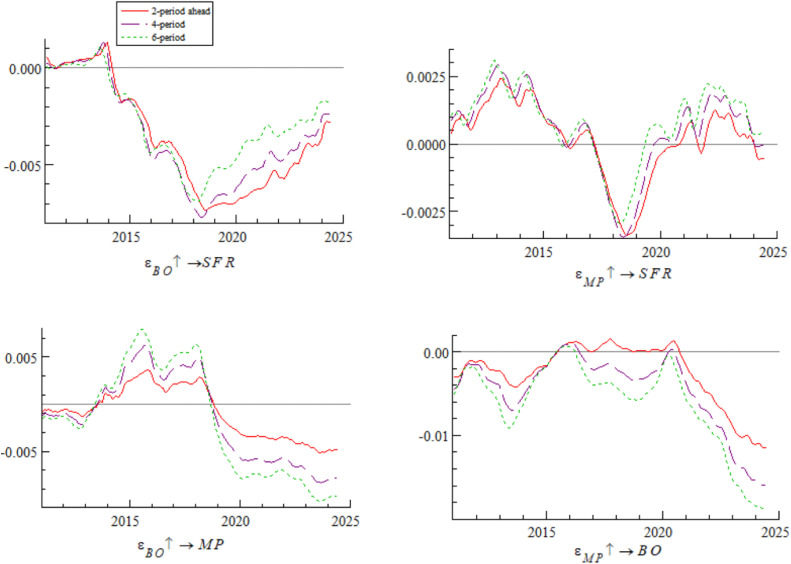
Robustness test of impulse responses under policy differences (Bond market opening).

This section uses China’s international investment position in debt securities as a proxy variable for bond market opening to conduct a robustness test. The impulse response results are shown in Figs 13 and 14. As can be seen from the figures, the overall impulse responses are consistent with the benchmark regression, confirming the robustness of the conclusions of this study.

### 5.2 Robustness test of systemic financial risks

In this section, robustness tests on the benchmark analysis conclusions of systemic financial risks are conducted from two dimensions—indicator replacement and method replacement—to exclude the interference of indicator selection bias and the subjectivity of weighting methods on the conclusions, and further verify the reliability of the core conclusions.

**Fig 15:**
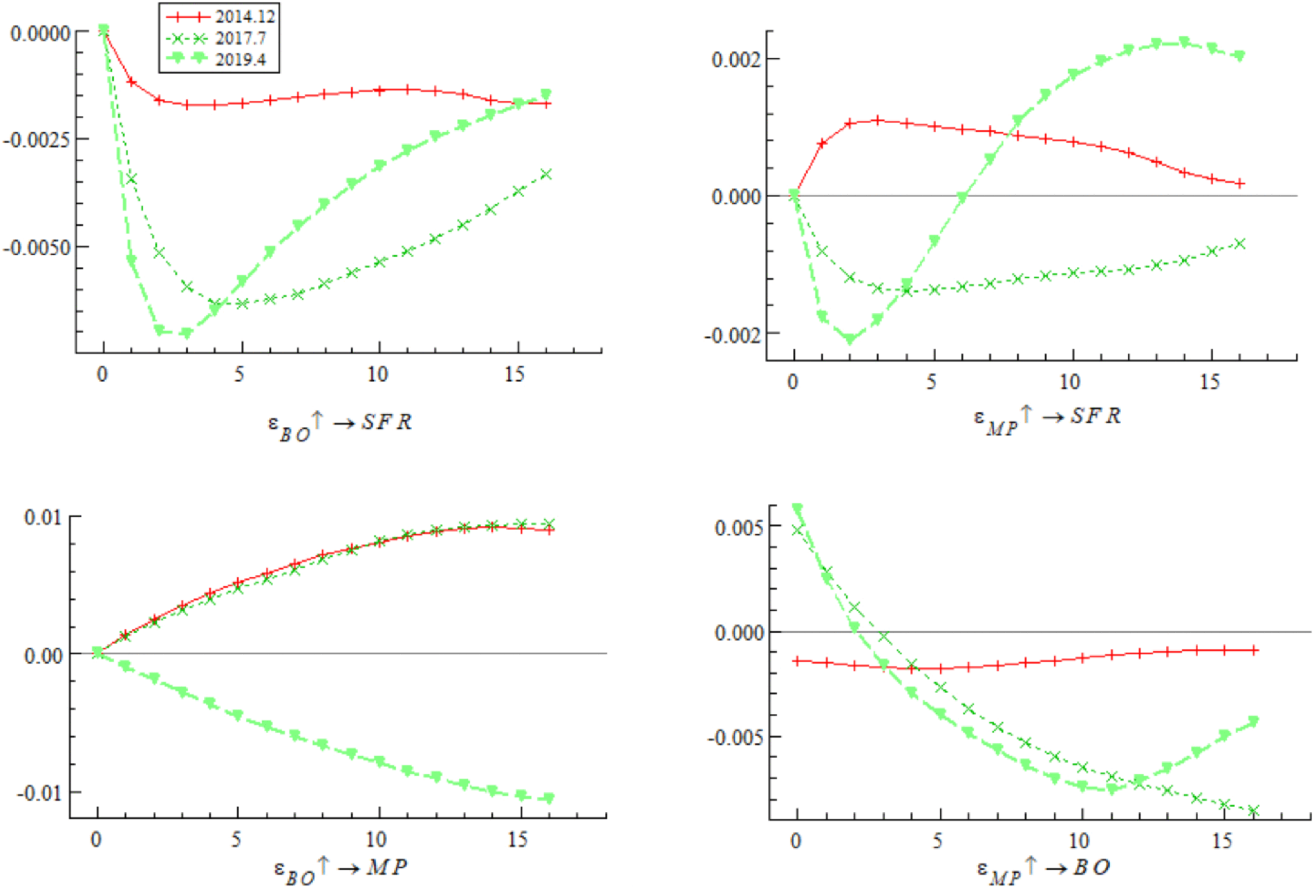
Robustness test of impulse responses at different ahead periods (Replacing systemic financial risks measurement indicators).

**Fig 16:**
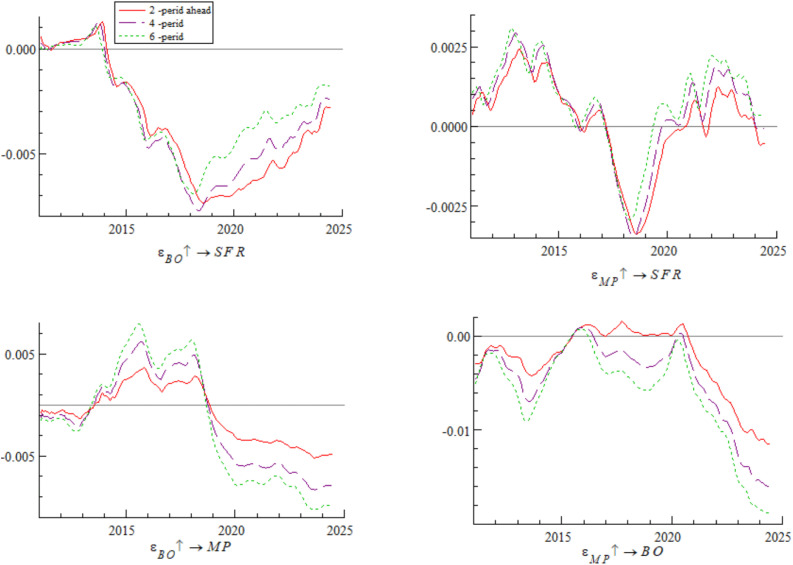
Robustness test of impulse responses under policy differences (Replacing systemic financial risks measurement indicators).

First, replacement verification is performed on the basic measurement indicators of the systemic financial risks index. Considering the representativeness and data availability of core indicators in each market, the Shenwan Real Estate Industry Index, Price-to-Book (P/B) ratio of financial institutions, CSI 300 Index, interbank 7-day pledged repo weighted interest rate, China Bond Composite Full-Price Index, and USD/CNY spot exchange rate are selected as alternative measurement indicators for the real estate market, financial institutions, stock market, money market, bond market, and foreign exchange market, respectively. The weighting method used in the benchmark analysis is adopted to reconstruct the systemic financial risks index, which is then used as the proxy variable for China’s systemic financial risks to conduct the robustness test. The corresponding impulse response results are shown in Figs 15 and 16. The test results indicate that after indicator replacement, the dynamic correlation characteristics between variables are generally consistent with those of the benchmark regression. This shows that the core conclusions of this study regarding the evolution law and influence mechanism of systemic financial risks are not affected by differences in indicator selection, exhibiting initial robustness.

**Fig 17:**
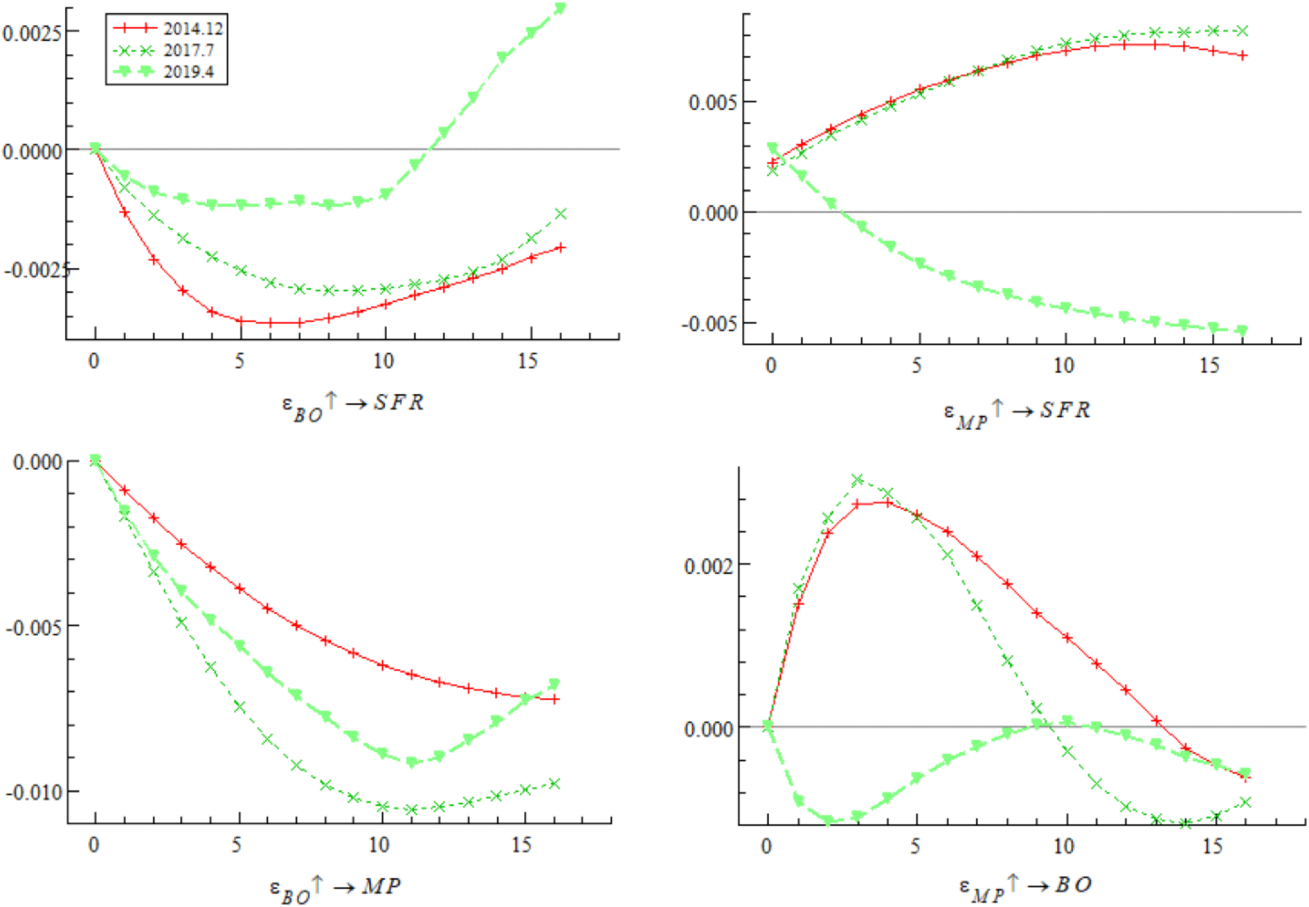
Robustness test of impulse responses at different ahead periods (Replacing systemic financial risks Weighting Method).

**Fig 18:**
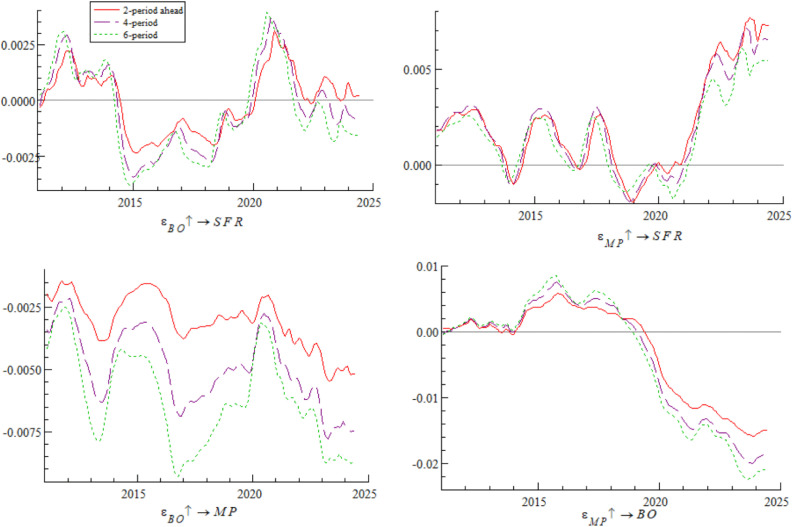
Robustness test of impulse responses under policy differences (Replacing Systemic financial risks Weighting Method).

Second, under the premise of maintaining consistency in the original indicator system, data frequency, and statistical scope, the weighting method of the systemic financial risks index is replaced to reduce the impact of the subjectivity of weighting methods on index construction. The Entropy Weight Method is used to replace the MWA-CRITIC weighting model in the benchmark analysis. The weights of each market indicator are recalculated to reconstruct the systemic financial risks index, and then the robustness test is conducted. The corresponding impulse response results are shown in Figs 17 and 18. The results demonstrate that after the replacement of the weighting method, the impulse responses are generally consistent with those of the benchmark regression, which further confirms the robustness of the core conclusions of this study and excludes the interference of weighting method selection on the research conclusions.

### 5.3 Robustness test of monetary policy

**Fig 19:**
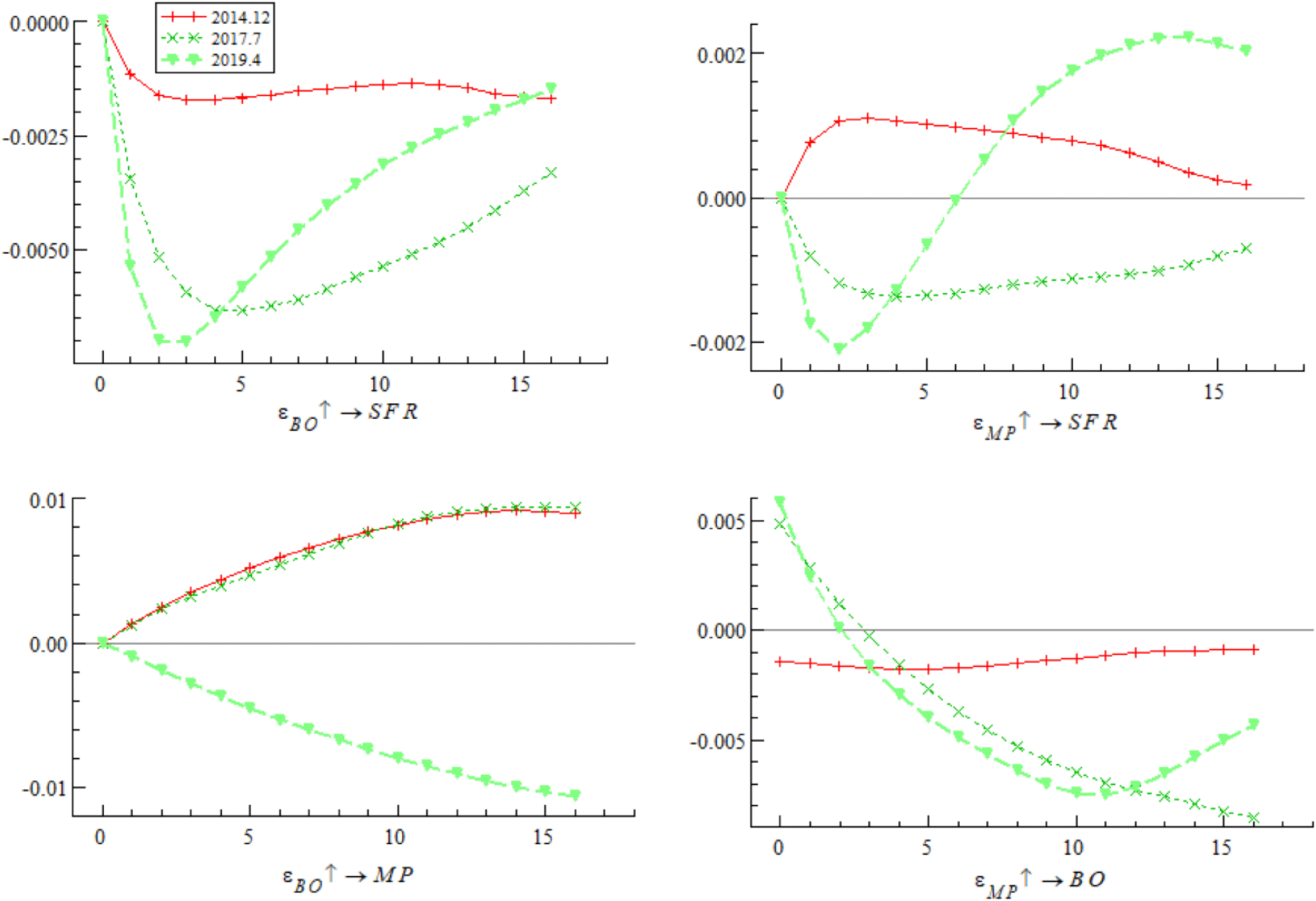
Robustness test of impulse responses at different ahead periods (Monetary policy).

**Fig 20:**
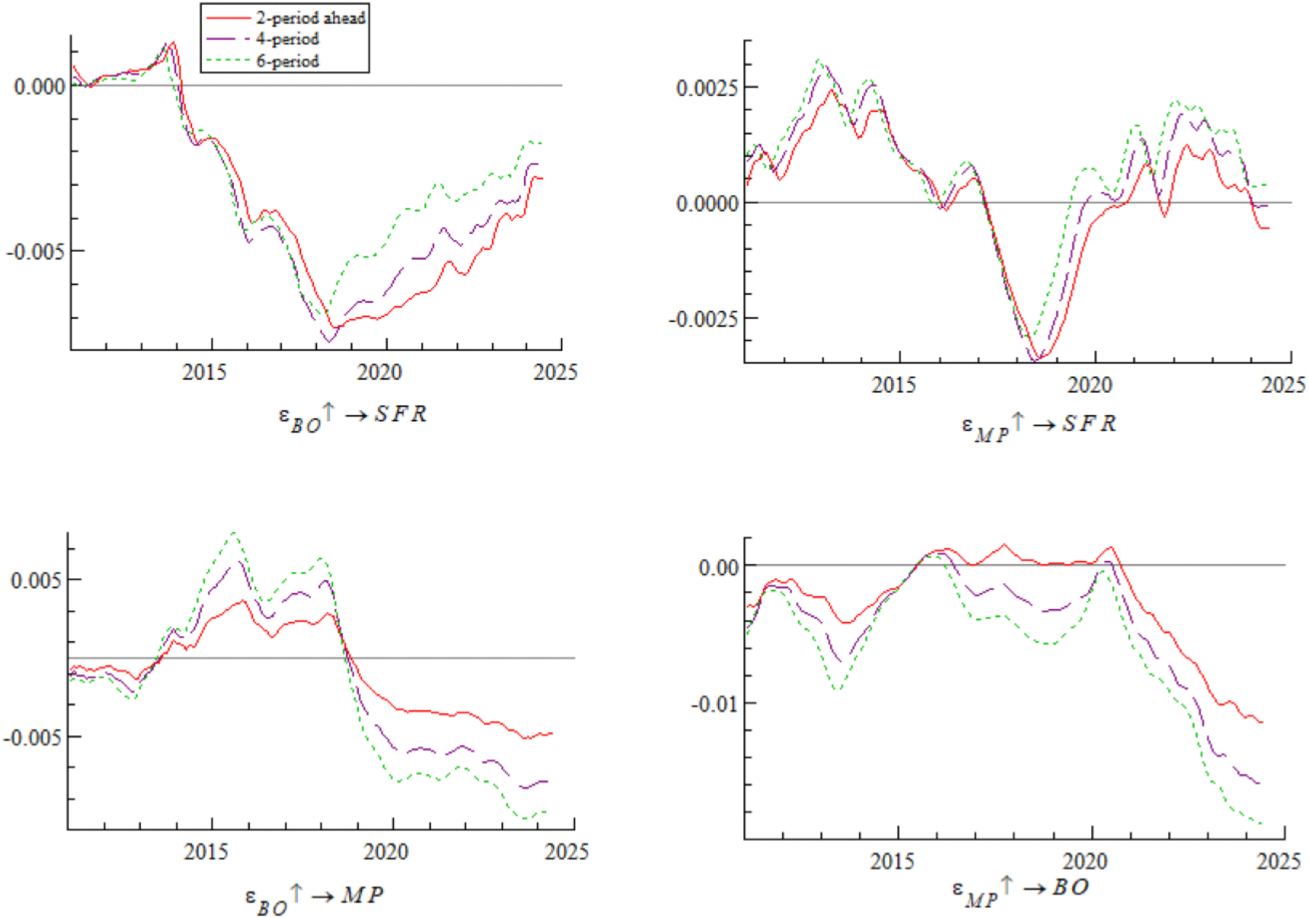
Robustness test of impulse responses under policy differences (Monetary policy).

In this section, the actively traded yield of 1-year Treasury bonds — closely related to monetary policy — is used as a proxy variable for monetary policy to conduct a robustness test. The impulse response results are shown in Figs 19 and 20. The results are consistent with the benchmark regression, confirming the robustness of the conclusions of this study.

## 6 Conclusion and implications

### 6.1. Conclusion

This study finds that, first, the impact of bond market opening on China’s systemic financial risks exhibits time-varying characteristics, forming a dynamic mechanism of “first promoting, then restraining, and then promoting again” through multiple pathways such as cross-border capital flows, cross-border risk transmission, and market structure optimization. Second, contractionary monetary policy exerts a restraining effect on systemic financial risks through the interest rate transmission mechanism, liquidity regulation, and adjustments in financial institutions’ behavior, but this effect shows diminishing marginal returns when faced with external structural shocks. Third, as the interest rate transmission mechanism continues to improve and the price-based monetary policy framework progresses steadily, the sustained regulatory effect of monetary policy on systemic financial risks has been significantly enhanced. Fourth, bond market opening and monetary policy exhibit significant interlinkage effects, which are subject to dual regulation from the progress of domestic institutional reforms and cross-border capital anomalies. Specifically, bond market opening suppresses the effectiveness of monetary policy through the linkage of domestic and foreign interest rates and the interest rate parity mechanism, and this suppressive effect is dynamic, closely related to the progress of domestic interest rate liberalization and cross-border capital anomalies. Meanwhile, monetary policy significantly promotes the opening of the bond market through the interest rate transmission mechanism and management of market expectations, but cross-border capital anomalies completely reverse this positive effect.

### 6.2. Implications

Based on the above empirical conclusions, to achieve the coordinated balance of “orderly bond market opening — precise monetary policy regulation — systemic financial risks prevention and control”, and combined with China’s financial opening reform process and international experience, the following targeted policy recommendations are proposed:

First, strengthen the supervision of cross-border capital flows and the risk hedging capacity of the bond market to fortify the risk defense line during opening. In the process of bond market opening, abnormal cross-border capital volatility and insufficient risk hedging tools are key factors inducing systemic risk, which require coordinated efforts from both regulatory early warning and tool support. On the one hand, the foreign exchange macro-prudential policy toolbox should be enriched and improved: cross-border bond investment should be incorporated into the macro-prudential management framework, and countercyclical adjustment factors should be set for short-term capital flows. Meanwhile, a dynamic risk early warning mechanism should be established, with the ratio of monthly cross-border capital net outflows to GDP and the concentration of foreign-held bonds as core early warning indicators. When these indicators hit the threshold, market expectations should be guided by issuing risk alerts and strengthening window guidance to prevent the impact of abnormal foreign capital volatility on systemic financial stability. On the other hand, it is necessary to accelerate the innovation and opening of the bond derivatives market: focus on launching tools such as long-term Treasury bond futures and Treasury bond options with a maturity of more than 10 years to fill the gap in long-term interest rate risk management. At the same time, relax the access threshold for foreign institutions to participate in interest rate swaps (IRS) and credit default swaps (CDS), and lift the position-matching restriction for foreign institutions to conduct risk hedging. This allows them to independently select hedging tools based on actual risk exposure, improve the management channels for interest rate and credit risks of RMB-denominated bonds, and reduce the probability of concentrated withdrawal of foreign capital due to unhedged risks.

Second, deepen the construction of institutional opening of the bond market to enhance its attractiveness for international investors’ allocation. Institutional opening is the core support for the bond market to attract long-term foreign capital and achieve sustainable opening, which requires a focus on rule alignment and mechanism improvement. At the legal framework level, revise the regulations and rules related to bond investment by overseas institutional investors, clarify the rights of foreign investors in information acquisition and default claims, establish a cross-border bond default resolution coordination mechanism, and reach mutual recognition agreements on judicial judgments with major economies to address the difficulty faced by foreign investors in protecting their rights. At the standard alignment level, promote the convergence of the bond credit rating system with international standards: require domestic rating agencies to disclose information such as internationally comparable ratings and risk factor weights in rating reports, and introduce international third-party rating agencies to conduct cross-validation. Meanwhile, accelerate the convergence of accounting standards, aligning rules such as bond interest calculation and impairment provision with the International Financial Reporting Standards (IFRS) to reduce the accounting costs of foreign investors. At the market mechanism level, improve the bond information disclosure system: require bond issuers to disclose the use of cross-border funds and changes in foreign investors’ positions on a quarterly basis. For bond-issuing enterprises in sensitive industries such as real estate and urban investment, additionally disclose risk indicators including cash flow coverage ratio and debt maturity structure. By enhancing market transparency, the attractiveness of RMB-denominated bonds to international long-term allocation funds is strengthened.

Third, advance interest rate marketization reform and the modernization transformation of the monetary policy framework to enhance the effectiveness of policy regulation. The degree of interest rate marketization and the maturity of the monetary policy framework directly determine the policy’s ability to regulate risks in an open environment, which requires breakthroughs in both transmission efficiency and framework transformation. In terms of interest rate marketization reform, further improve the interest rate corridor mechanism and strengthen the transmission signal of policy interest rates to market interest rates. In terms of the transformation of the monetary policy framework, accelerate the shift from a quantity-based to a price-based regulatory framework, reduce reliance on quantity-based tools such as credit scale and foreign exchange reserves accumulation, and use more price-based tools such as open market operations (OMO) and Medium-term Lending Facility (MLF) to adjust market liquidity. Meanwhile, construct a two-pillar regulatory model combining the interest rate corridor and macro-prudential policy, incorporate factors such as bond market liquidity and cross-border capital flows into policy considerations, and resolve the dilemma of diminished monetary policy effectiveness under external shocks by precisely adjusting the level of policy interest rates.

Fourth, establish a dynamic balance mechanism between bond market opening and financial system stability to achieve the integration of opening and security. The opening of the bond market must match the carrying capacity of the financial system, which requires the establishment of a balance mechanism among dynamic assessment, coordinated regulation, and countercyclical adjustment. First, establish a dynamic assessment system for bond market opening: regularly assess the impact of opening on financial stability from three dimensions — foreign capital structure, risk resilience, and policy adaptability — and adjust the pace of opening based on the assessment results. For example, when the proportion of long-term foreign capital exceeds the set threshold, suspend the launch of new opening measures. Second, establish a coordination framework between monetary policy and bond market opening: incorporate key variables such as cross-border capital flows, exchange rate fluctuations, and the international interest rate environment into the policy decision-making model, and dynamically optimize the policy mix. For instance, during the Federal Reserve’s interest rate hike cycle and the inversion of China-U.S. interest rate spreads, adopt a combined operation of reducing the required reserve ratio and raising the foreign exchange deposit reserve ratio — this not only releases liquidity to stabilize the market but also curbs capital outflows to ease exchange rate pressure. During periods of global liquidity easing, use a combination of interest rate hikes and expanding the quota of Bond Connect to attract foreign capital inflows while preventing asset bubbles. Finally, give full play to the countercyclical adjustment role of macro-prudential policy: include the scale of foreign-held bonds and the leverage ratio of cross-border bond financing into the Macro Prudential Assessment (MPA) evaluation, implement differentiated capital requirements for financial institutions with excessive cross-border risk exposure, and guide them to reasonably control the scale of cross-border business. This fortifies the security barrier of the national financial system while steadily advancing the opening of the bond market.
